# The tip of the iceberg: extraordinarily high diversity while examining two infralittoral nematode communities on Okinawa-jima Island, Japan, using morphology and DNA barcoding

**DOI:** 10.7717/peerj.19757

**Published:** 2025-07-30

**Authors:** Marilyn Carletti, Nuria Viñuela Rodríguez, Gaia Rossetti, Virginia Rossi, Bryan Gabriel Pulido Tan, James Davis Reimer

**Affiliations:** 1 Molecular Invertebrate Systematics and Ecology Lab, Graduate School of Engineering and Science, University of the Ryukyus, Nishihara, Okinawa, Japan; 2 Marche Polytechnic University, Ancona, Italy; 3 University of the Ryukyus, Graduate School of Engineering and Science, Nishihara, Okinawa, Japan; 4 Tropical Biosphere Research Center, Graduate School of Engineering and Science, University of the Ryukyus, Nishihara, Okinawa, Japan

**Keywords:** Infralittoral, Biodiversity, Coral reefs, Ryukyu archipelago, Roundworms

## Abstract

**Background:**

Nematodes are among the most diverse and abundant metazoans in aquatic habitats, contributing significantly to global biodiversity. Despite their abundance and importance, the presumed number of undescribed species is high and their diversity is often underestimated.

**Methods:**

In this research, sediment samples were collected from three microhabitats (bare sand, seagrass, coral) in two sites around Okinawa-jima Island in subtropical southern Japan. Nematode specimens were obtained by filtering the sediment and were then used to determine meiofaunal assemblages with morphology and molecular methods at the two sites and to compare them with environmental variables.

**Results:**

The results showed an overwhelmingly high biodiversity of nematofauna with both methods. The morphological identification of free-living nematodes was partly supported by molecular analyses, with the results varying more regarding less common taxa. The discrepancies between different methods may be due to low success of DNA amplifications, high nucleotide variability, and overestimation of congeneric specimens. We observed that coral reef habitats clearly differed from nearby sand and seagrass beds in terms of nematode genus-level assemblages. We identified at least 10 orders and 38 genera of nematodes from our samples that only span two different sites, and it is highly likely these samples include undescribed taxa. Our results strongly suggest that coral reefs and neighboring areas are hot-spots for nematode diversity, at least around Okinawa-jima Island if not also in other coral reef regions.

## Introduction

Free-living nematodes are among the most diverse and abundant metazoans in aquatic habitats (Appeltans et al., 2012; Lambshead, 2004). Nematodes contribute substantially to worldwide biodiversity, especially in marine sediments, where they may reach very high abundances (Grassi et al., 2022; Moens et al., 2013; Semprucci et al., 2018).

Nematode identification is particularly challenging and it is usually possible only by trained taxonomic experts (Bogale, Baniya & Di Gennaro, 2020), which are unfortunately reported as decreasing in number (Nisa, Tantray & Shah, 2022). Because of the presumed high number of undescribed nematode species and the overall lack of current research, participation from students and researchers is limited (Appeltans et al., 2012; Ridall & Ingels, 2021). With the decline of the number of taxonomists (Boufahja et al., 2010; Ridall & Ingels, 2021; Nisa, Tantray & Shah, 2022) and the concurrent rise in molecular methods, DNA barcoding now plays a significant role in nematode diversity research. Most nematode DNA barcoding studies initially used the cytochrome c oxidase sub-unit 1 (COI) gene fragment because of its ubiquity and suitability for many organisms. The COI gene has been successfully used in the barcoding of marine nematodes to resolve taxonomic relationships among closely related and/or cryptic species (Blouin et al., 1998; Derycke et al., 2005). However, this marker has been shown to have low amplification success overall for nematodes as a result of mutations in primer-binding regions (Blaxter et al., 1998; De Ley et al., 2005; De Ley, 2006). More recent barcoding studies (Armenteros et al., 2014; Avó et al., 2017; Bik et al., 2010a; Bik et al., 2010b; Chariton et al., 2014; Derycke et al., 2010; Pereira et al., 2010; Schenk, Kleinbölting & Traunspurger, 2020) that investigated meiofaunal organisms including nematodes have suggested the use of nuclear 18S small subunit (SSU) ribosomal DNA (18S rDNA) and 28S large subunit (LSU) ribosomal DNA (28S rDNA). Even though no standardized gene for DNA barcoding is available for marine nematodes, the accumulation of nematode 18S and 28S nuclear ribosomal DNA (rDNA) sequences in public databases reflects their utility and widespread use in molecular phylogenetic studies (Avó et al., 2017; Blaxter et al., 1998; Kumar, Sen & Bhadury, 2014; Litvaitis et al., 2000; Nadler, 1992; Nadler et al., 2007; Pereira et al., 2010).

Okinawa-jima Island is the largest island of the Ryukyu Archipelago, which spans the region between southern mainland Japan to Taiwan. Okinawa-jima Island is situated in an area with high marine biodiversity that is under threat from numerous local anthropogenic impacts (Roberts et al., 2002) including terrestrial runoff (Shilla et al., 2013), pollution (Takeuchi, 2023), and coastal development (Masucci & Reimer, 2019). Despite these threats, marine biodiversity research in the surroundings of Okinawa-jima Island, as in many places in the world, remains unbalanced, with a clear need for more research on so-called “minor” taxa such as nematodes, as well as more research on marine ecology and conservation also being critically needed (Reimer et al., 2019).

Until now, the diversity of marine nematodes of Okinawa-jima Island has been little studied and only a few reports are available (*e.g.*, Leduc & Sinniger, 2018; Leduc, 2022). The aim of this study was to help address this research gap, and we assessed the marine diversity of the phylum Nematoda from two shallow coral reef sites around Okinawa-jima Island, Japan, by comparing the results obtained from morphological and molecular analyses (18S rDNA and 28S rDNA sequences) of single-picked nematode specimens in accordance with current research standards. The aim of the present study is to evaluate nematode diversity and their phylogenetic relationships in a little-studied area, in consideration of their importance for potential downstream use as bioindicators of coral reefs. The results will help establish a baseline for nematode diversity research for coral reefs around Okinawa-jima Island, and help generating new sequences for genera not registered in the common databases.

## Materials & Methods

### Sites and sampling strategy

**Figure 1:**
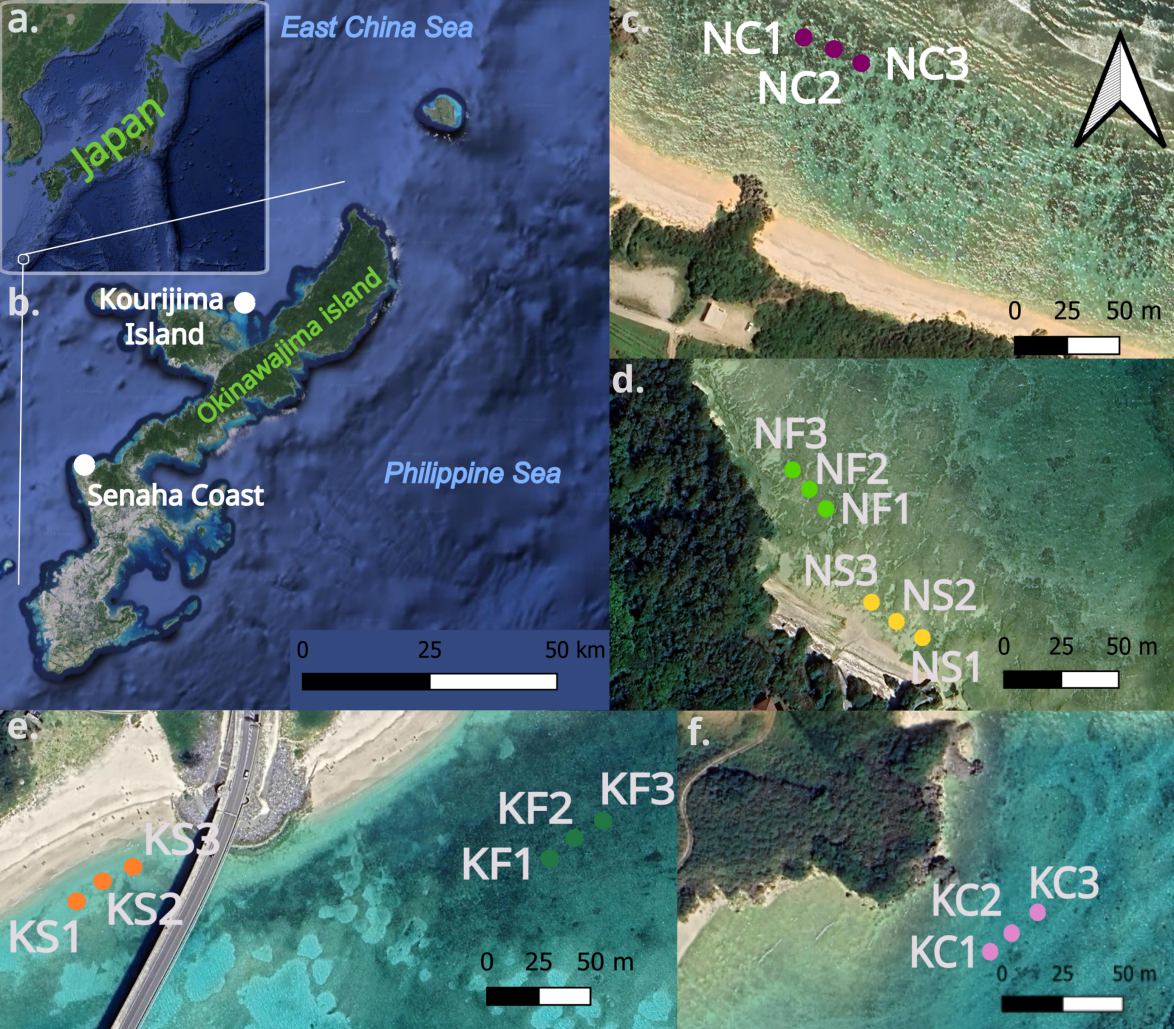
Map of Okinawa-jima Island with sampling sites in this study. Map of Japan and Okinawa-jima Island (A, B) Transects at Senaha Coast (C, D) and at Kouri-jima Island (E, F). The images were obtained through open source data on QGIS.

Sediment samples were collected in the infralittoral area of two sites around Okinawa-jima Island (Japan, Figs. 1A, 1B.); Senaha Beach (26.4247°N, 127.7342°E, Figs. 1C, 1D) and Kouri-jima (26.7067°N, 128.0181°E, Figs. 1E, 1F). Samples were collected during low tide at depths between 0.8 m and 2.1 m (Table 1) between October 15 to November 11, 2022. These two locations were selected because their micro-habitat complexity over a small area was ideal to make comparisons within each site; with seagrass, bare sand, and coral outcrops all within 800 m of each other. Latitude and longitude coordinates were taken by using a handheld GPS device (Meiri, 2018; Table 1). Figure 1 was created using QGIS (Version 3.36.2, QGIS Development Team, 2024), using the QuickMapServices plugin with Google Maps (https://maps.google.com) satellite layer.

At each microhabitat within both sites, three sediment samples were collected every 10 m each from sea grass-dominated areas, bare sand areas, and coral outcrops (Figs. 1C, 1D, 1E; Table 1), making a total of 9 collected sediment samples per site and 18 overall. Sediment samples were used for morphological and molecular analyses and were obtained by pushing the manual corer tube (four cm Ø, 10 cm H) vertically into the sediment. Sediments were then fixed into a mixture of 4% formalin and rose Bengal or 80% ethanol, and stored at −4 °C.

All sediment samples were washed and processed using colloidal silica Ludox^®^ (DuPont) HS 40 as a high-density solution (Burgess, 2001). The sediment was then filtered through a two mm mesh sieve to eliminate coral rubbles and rocks. Each sample was divided and transferred into four Falcon^®^ tubes that were filled with a mix of two parts of Ludox^®^ and three parts of distilled water, centrifuged at 3,000 g for 10 min and washed again. Finally, the supernatant was filtered with a 45 µm sieve. This process was repeated three times to maximize the number of nematode specimens collected.

**Table 1:** Coordinates of sampling locations around Okinawa-jima Island in this study.

**Site**	**Habitat**	**Replicate code**	**Latitude**	**Longitude**	**Depth (m)**	**Date**	**Observations**
Senaha	Sand 1	NS1	26.42474	127.73393	1.0	10/21/2022	
Senaha	Coral 1	NC1	26.42667	127.73485	1.8	10/22/2022	Coral bleaching observed
Senaha	Seagrass 1	NF1	26.42483	127.73396	1.0	10/21/2022	
Senaha	Sand 2	NS2	26.42477	127.73385	1.0	10/21/2022	
Senaha	Coral 2	NC2	26.42669	127.73493	2.1	10/22/2022	Coral bleaching observed
Senaha	Seagrass 2	NF2	26.42487	127.73387	1.0	10/21/2022	
Senaha	Sand 3	NS3	26.42492	127.73378	1.0	10/21/2022	
Senaha	Coral 3	NC3	26.42678	127.73490	2.0	10/22/2022	*Acropora* spp. present
Senaha	Seagrass 3	NF3	26.42493	127.73380	1,0	10/21/2022	
Kouri	Sand 1	KS1	26.69428	128.02122	1.1	15/10/2022	
Kouri	Coral 1	KC1	26.69958	128.02738	0.8	08/11/2022	*Acropora* spp. present
Kouri	Seagrass 1	KF1	26.69437	128.02314	1.5	15/10/2022	
Kouri	Sand 2	KS2	26.69424	128.02113	1.0	15/10/2022	
Kouri	Coral 2	KC2	26.69965	128.02750	1.0	08/11/2022	*Acropora* spp. present
Kouri	Seagrass 2	KF2	26.69441	128.02322	1.4	15/10/2022	
Kouri	Sand 3	KS3	26.69419	128.02105	1.0	15/10/2022	
Kouri	Coral 3	KC3	26.69971	128.02758	1.0	08/11/2022	*Acropora* spp. present
Kouri	Seagrass 3	KF3	26.69446	128.02329	1.3	15/10/2022	

### Environmental data collection

To reduce potential biases due to environmental factors at the sites, environmental variables and anthropic pressures and impacts were measured. Additional sediment and seawater samples were collected from each habitat. Seawater was taken using a simple bucket (24 × 24 cm) from the surface in each location and the sediment was collected with cylindrical manual corers (4 cm Ø, 15 cm H) at the seafloor (Table 1). We analyzed water quality parameters using a RINKO probe (data for temperature, salinity, conductivity, dissolved oxygen, turbidity, EC25, density, chlorophyll fluorescence, chlorophyll-a; Table 2). From the collected sediment, we conducted a particle size analysis (PSA) on each sample using a granulometric analyser (Particle Image Analyzer; JASCO Labs FF-30Micro) to determine granulometry ISO mean and the percentages of silt/clay (*ca* < 63 µm), sand (*ca* 63–2,000 µm), and gravel (*ca* > 2,000 µm), as defined by Blott & Pye (2012). These analyses were performed one time per replicate, and thus three times for each microenvironment for each site (total *n* = 18).

**Table 2:** Means of the environmental variables in each site and habitat.

** **	** **	**Senaha_seagrass**	**Senaha_sand**	**Senaha_coral**	**Kouri_seagrass**	**Kouri_sand**	**Kouri_coral**
***In-situ* evaluation**	Date of sampling	21/10/2022	21/10/2022	22/10/2022	15/10/2022	15/10/2022	08/11/2022
** **	Locality	Senaha Beach	Senaha Beach	Senaha Beach	Kouri-jima	Kouri-jima	Kouri-jima
** **	Tide at sampling	Low	Low	Low	Low	Low	Low
**Granulometry machine measures**	ISO inner diameter mean (µm)	294.82	450.75	2,263.63	1,466.67	767.50	1,168.07
** **	Silt/clay	0.37	0.00	0.00	0.00	0.00	0.00
** **	Sand	98.37%	97.76%	73.50%	82.37%	92.80%	92.80%
** **	Gravel	1.26%	8.90%	25.38%	17.63%	7.20%	7.20%
**RINKO probe**	Temp (deg C)	28.43	28.43	29.08	28.88	28.96	28.19
**data**	Sal (PSU)	32.69	32.69	33.40	28.90	20.08	24.16
** **	Cond (mS/cm)	53.35	53.35	55.24	48.20	34.77	40.49
** **	EC25 (uS/cm)	49,600.86	49,600.86	50,692.26	44,406.31	31,978.81	37,836.11
** **	Density (kg/m^3^)	1,020.52	1,020.52	1,020.94	1,017.53	1,010.91	1,014.21
** **	Chl-Flu, (ppb)	0.77	0.77	0.46	0.34	0.43	0.34
** **	Chl-a (ug/l)	0.77	0.77	0.46	0.34	0.43	0.34
** **	Turb-M (FTU)	2.63	2.63	1.70	1.13	2.08	1.38
** **	DO (%)	132.03	132.03	118.60	101.91	110.82	102,16
** **	Weiss-DO (mg/l)	8.52	8,52	7.56	6,564.79	7,596.66	6,944.17

Additionally, we used the “DiBattista scale” (DiBattista et al., 2020) to estimate the level of cumulative anthropic pressure and impact present for both sites investigated. DiBattista et al. (2020) analyzed the impact of natural and anthropogenic pressures on selected sampling sites in Okinawa-jima Island *via* a point-based assessment system in order to rank sites based on cumulative anthropogenic impacts. Criteria considered in this scale included distance from shore, distance from heavily populated areas, freshwater input, fishing pressure, coastal development, presence of human recreation area, hermatypic coral cover, and other notable pressures. Scored sites were then grouped into low (score = 10 to 7), medium (score = 7 to 4), or high (score = 4 to 0) anthropogenic pressure groups.

### Morphological analyses

A total of 100 nematodes (or all available individuals if fewer than 100) were randomly selected from each replicate using a stereomicroscope (LEICA S8AP0). The nematodes obtained were mounted on permanent slides by using a simplified version of the Seinhorst (1962) method and identified with an optical microscope (Nikon ECLIPSE 80i), using a maximum magnification of 100X. For identification, we used the pictorial keys from Platt & Warwick (1983), Platt & Warwick (1988), Warwick, Platt & Somerfield (1998), Wieser (1953), the World Database of Nematodes Nemys (Nemys, 2024; https://nemys.ugent.be/), and papers including the most up-to-date information relating to the taxa found (Ahmed, Boström & Holovachov, 2020; Fadeeva, Mordukhovich & Zograf, 2016; Kotta & Boucher, 2001; Leduc, 2013; Leduc & Sinniger, 2018; Leduc & Zhao, 2021; Muthumbi & Vincx, 1999).

The data obtained through morphological analyses were examined using the software R (R Core Team, 2025). The Chao 1 (Chao, 1984) and Shannon (Shannon, 1948) indices were calculated and confronted (Oksanen et al., 2025; Wickham, 2016; Wickham, 2025)

Additionally, a multivariate analysis of variance based on permutations (PERMANOVA; Anderson, 2001) was performed on transformed data to calculate the significant variance in nematode assemblages among sites and habitats. A similarity of percentage (SIMPER) analysis was used to determine which taxa influenced the spatial distribution, and a PERMDISP analysis was also conducted to test the null hypothesis among the analyzed environmental parameters. These analyses were performed with PRIMER-e 7 + PERMANOVA (Anderson, 2001).

The environmental data (depth, DiBattista scale information, ISO inner diameter mean, silt/clay%, sand%, gravel%, as well as temperature, salinity, conductibility, chlorophyll-a and dissolved organic matter of seawater) were imported and transformed using the function “(x-mean)/stdev”. To visualize possible differences between the structure of the communities and to clarify the relationship between environmental data and abundance, a Distance-based multivariate multiple regression (DistLM), a principal component analysis (PCA), and a non-metric multidimensional scaling (nMDS) analysis were performed on the selected database. The nMDS analysis was performed using Bray-Curtis dissimilarity and log-transformation to reduce the influence of abundant taxa. Statistical analyses and graphics were obtained using the software Past 4.03 (Paleontological Statistics Software Package for Education and Data Analysis; Hammer, Harper & Ryan, 2001).

### DNA extraction, PCR conditions, and sequencing

Nematode specimens were randomly picked from each replicate with the same method used for the morphological analyses. The organisms were individually picked using a sterile syringe, placed on a microscopy slide, rapidly identified and finally transferred singularly into 1.5 ml tube. The slides were cleaned between every specimen using bleach and heat sterilization by flaming and rinsed with ethanol to remove any residues from the surface. We used the commercial kit Qiagen DNeasy Blood and Tissue Kit (QIAGEN, Hilden, Germany) following the manufacturer’s standard protocol (Haendiges et al., 2020) for DNA extraction. To avoid an initial low percentage of successful extractions, we repeated the final elution step in the AE buffer two times for a total volume of 50 µl of DNA.

To assess trends and standards in free-living marine nematode phylogenetic research, we searched literature using Google Scholar and ISI Web of Science in January 2024. We used the key words; “free-living”, “marine”, “nematode” and “phylogeny” or “phylogenetic trees” or “evolution” and excluded “freshwater”, “soil”, “terrestrial” and “parasite” to avoid including research conducted only on parasitic nematodes or free-living terrestrial or freshwater nematodes. We found seven peer-reviewed publications matching our criteria and summarized them to highlight markers and primers commonly used in phylogenetic analyses until now (Supplemental Information 8; Armenteros et al., 2014; Avó et al., 2017; Bik et al., 2010a; Bik et al., 2010b; Derycke et al., 2010; Kumar, Sen & Bhadury, 2014; Pereira et al., 2010). Based on these results, for this study we chose to amplify 18S rDNA and 28S rDNA, as they have been proven to be effective for a wide range of different taxa within the phylum Nematoda.

Target gene fragments were then amplified *via* PCR. Each 25 µl PCR reaction for the two markers consisted of 2.5 µl of 10X Standard Taq Reaction Buffer, 0.5 µl of 10 mM dNTPs, 0.5 µl of forward primer, 0.5 µl of reverse primer, 0.125 µl of Taq DNA polymerase, 1.0 µl of MgCl, 18.375 µl of ultra-pure RNA-free water and 1.5 µl of raw DNA.

To amplify 18S rDNA, we used the primer pair Nem18SF (5′-ATTCCGATAACGARCGA GAC-3′)/Nem18SR (5′-CCGCTKRTCCCTCTAAGAAGT-3) (Wood et al., 2013) and for 28S rDNA, D2A (5′ ACAAGTACCGTGAGGGAAAGT3′) and D3B (5′ TGCGAAGGAACCAGCTACTA 3′) (Nunn, 1992; Zhao et al., 2008). The optimized PCR conditions were an initial denaturation of 5 min at 94 °C, followed by 40 cycles of 94 °C for 30 s, 54 °C for 30 s, 72 °C for 1 min, then followed by a final extension of 10 min at 72 °C. PCR products were visualized on a 1.2% agarose gel, and successful amplifications were purified using exonuclease and shrimp alkaline phosphatase digestion, and sent for Sanger sequencing in both directions to FASMAC (Kanagawa, Japan).

#### Phylogenetic analyses

New sequences obtained were edited in Geneious (Geneious Prime 2023.2; Kearse et al., 2012) using chromatograms to assess their quality. Short sequences (<500 bp) and/or those of low sequence quality (<80%) were discarded. Forward and reverse sequences were assembled and trimmed with the same software. We used BLAST (Basic Local Alignment Tool; Altschul et al., 1990) to search individually for nucleotide sequence homology and MEGA11 (Molecular Evolutionary Genetics Analysis version 11; Tamura, Stecher & Kumar, 2021) to create a reference database of the taxa identified. In addition, previously reported 18S rDNA (*n* = 66) and 28S rDNA (*n* = 16) sequences of marine nematodes from GenBank were included in our respective databases (Supplemental Information 1). All sequences were aligned using MUSCLE (MUltiple Sequence Comparison by Log-Expectation; Edgar, 2004) as implemented in MEGA11 with default parameters and the ends of the resulting alignments were trimmed.

DnaSP v6.12.03 (Rozas et al., 2017) was used to obtain a list of the potential haplotypes among all sequences amplified (Supplemental Information 2), which were uploaded on MEGA11 (Tamura, Stecher & Kumar, 2021) to select the best nucleotide substitution model (GTR+G for the 18S sequences and GTR+G+I for the 28S sequences), align the sequences, and create a maximum likelihood tree (bootstrap substitution rate = 1,000).

In addition, gene trees were reconstructed with the same dataset by applying Bayesian Inference (BI) on MrBayes version 3.2.7 (Ronquist et al., 2012), through the CIPRES Science Gateway (Miller, Pfeiffer & Schwartz, 2010).

The BI trees were re-rooted in order to separate the two classes of the phylum Nematoda (Chromadorea and Enoplea) and the outgroup pruned to have a clearer view of clades (see original trees in Supplemental Information 3–6). The obtained trees were visualized with FigTree version 1.4.4 (Rambaut, 2018) and edited with Inkscape version 1.3.2 (Inkscape Project, 2020, https://inkscape.org/).

### Comparison of methods

We used a Venn diagram, elaborated with R (R Core Team, 2025) and using indications from Gao (2023) and Yan (2023), to visualize the similarity and efficiency among our classic morphological identification and DNA barcoding of the 18S rDNA and 28S rDNA markers.

## Results

### Granulometry and environmental variables

All sediment samples (*n* = 6) were characterized by the dominance of sand (73.50%–98.37%), followed by gravel and clay fractions, with a mean grain diameter ranging from 294.82 µm in Senaha_seagrass to 2,263.63 µm in Senaha_coral (Table 2). On average, seawater temperatures were stable, ranging from 28.19 °C to 29.08 °C. Salinity, conductivity and chlorophyll-a level were higher in Senaha habitats than at Kouri-jima (Table 2).

### Morphological results

**Figure 2:**
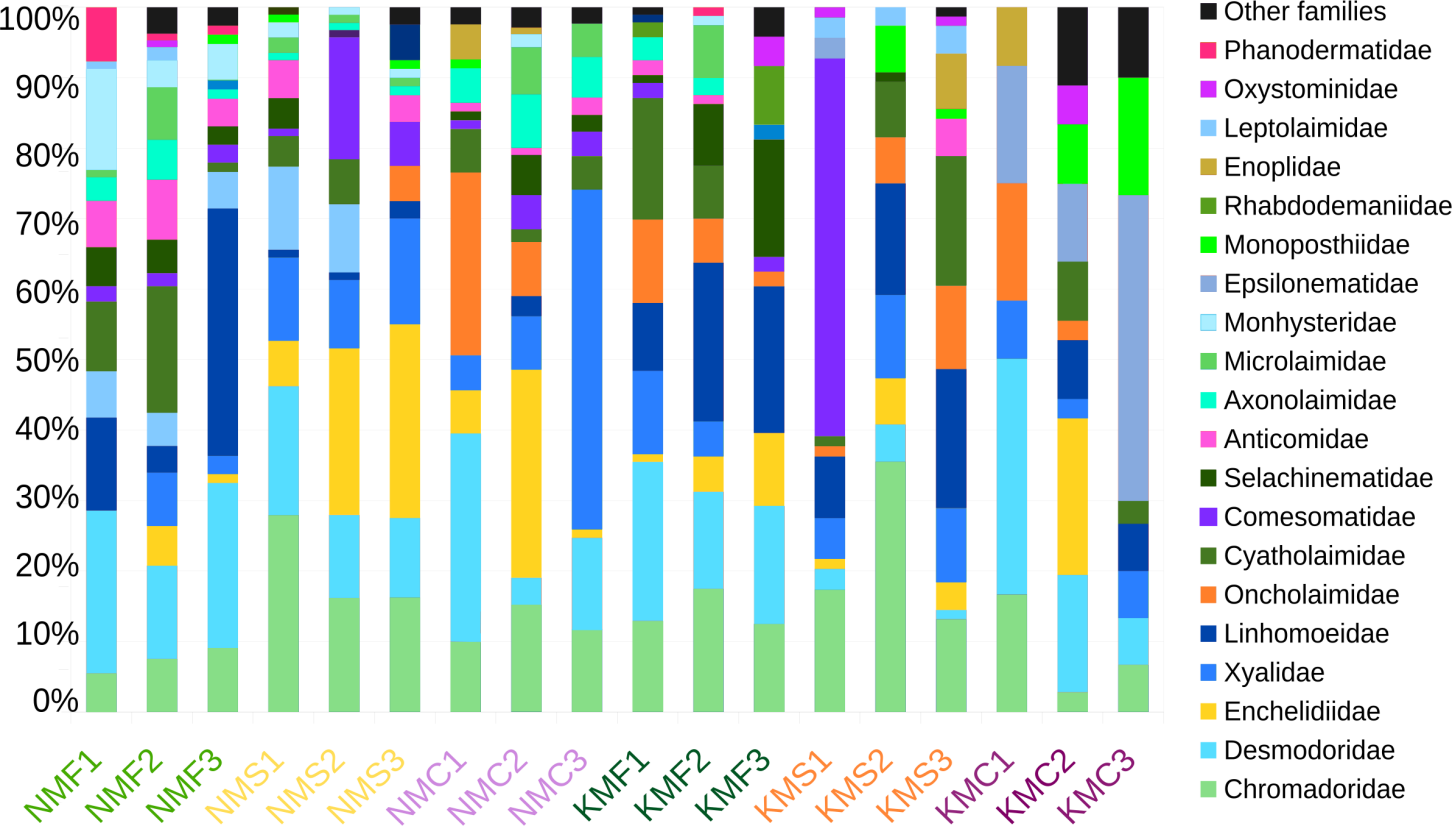
Abundance percentage nematode families. N, Senaha; K, Kouri; M, morphology; F, seagrass; S, sand; C, coral. Numbers refer to replicate.

Focusing on the phylum Nematoda, 24 families were found, with the top five families in terms of abundance (Chromadoridae, Desmodoridae, Cyatholaimidae, Linhomoeidae and Oncholaimidae) contributing more than 60% of the total nematode numbers (Fig. 2). The total dataset of all samples consisted of 104 nematode taxa, of which 80 were identified to the level of genus, one to subfamily and 23 to family (Supplemental Information 7). The three most common taxa in the class Enoplida were *Pareurystomina* sp. (4.38%, *n* = 60), *Viscosia* sp. (3.58%, *n* = 49), and Enchelidiidae indet. (2.55%, *n* = 35), while in the class Chromadorea, we found Linhomoeidae indet. (5.99%, *n* = 82), *Vasostoma* sp. (4.38%, *n* = 60) and *Daptonema* sp. 2 (3.94%, *n* = 54). *Daptonema* sp. 2, *Pareurystomina* sp. and Enchelidiidae were the most common taxa in Senaha_sand; *Daptonema* sp.1, *Pareurystomina* sp. and *Viscosia* sp. in Senaha_coral; Linhomoeidae, *Paracanthonchus* sp. and *Didelta* sp. in Senaha_seagrass; *Vasostoma* sp., Linhomoeidae and *Prochromadorella* sp. in Kouri_sand, *Epsilonema* sp. Monoposthiidae sp. and *Eurystomina* sp. in Kouri_coral and Linhomoeidae, *Molgolaimus* sp. and *Parapinnanema* sp. in Kouri_seagrass.

**Figure 3:**
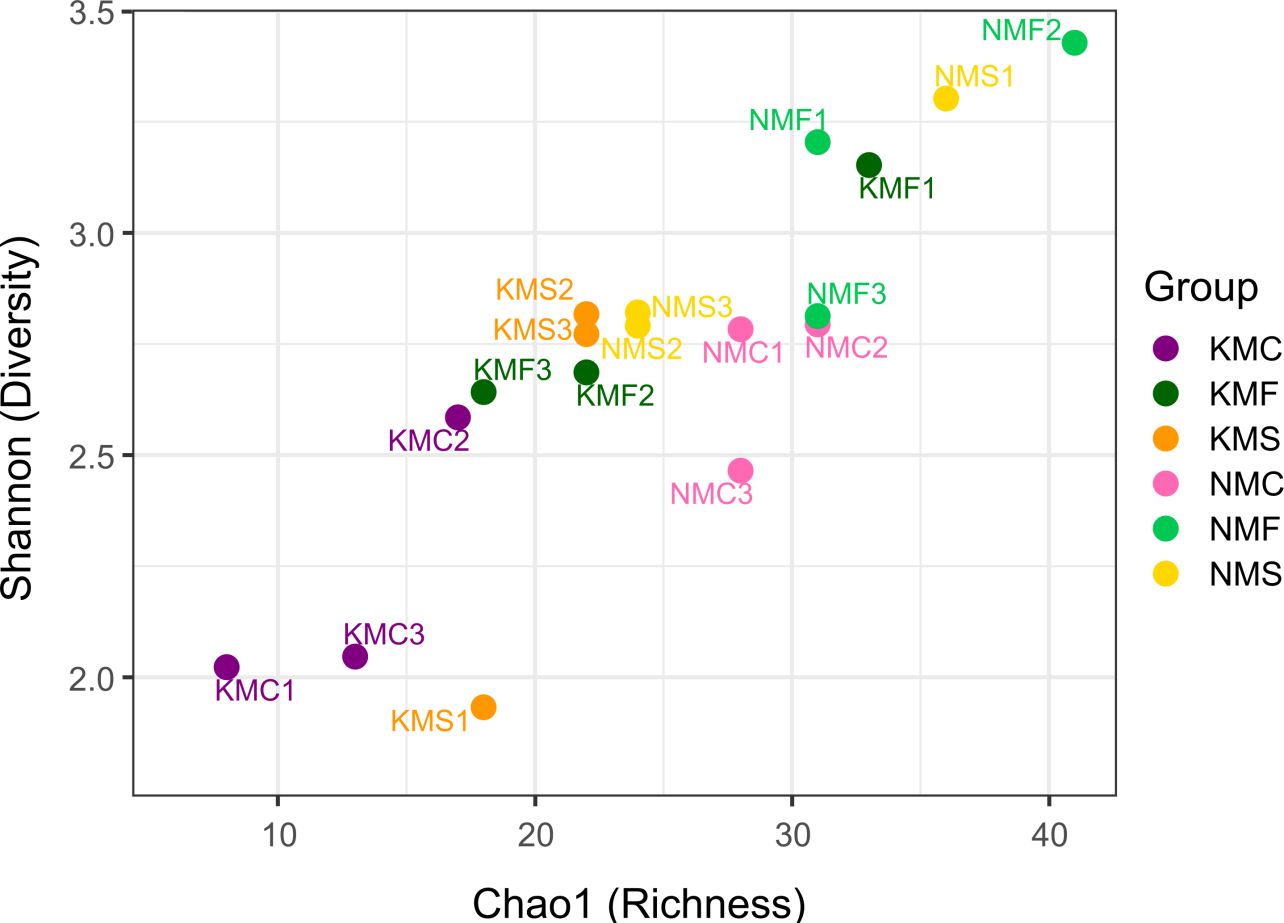
Chao1 Index and Shannon Index scatter plot. N, Senaha; K, Kouri; M, morphology; F, seagrass; S, sand; C, coral. Numbers refer to replicate.

The scatter plot of Chao1 and the Shannon indices are represented in Fig. 3. The Chao1 values in our samples ranged from 8 (KMC1) and 41 (NMF2), indication of an overall substantial difference in terms of species richness between samples. NMF2 (Chao1 = 41), NMS1 (Chao1 = 36) and KMF1 (Chao1 = 33) were the richest samples, as they included more rare taxa, while KMC1 (Chao1 = 8), KMC2 (Chao1 = 13), KMC3 (Chao1 = 17) were the least rich. The Shannon values, instead, ranged from 1.93 (KMS1) to 3.43 (NMF2), demonstrating a significant variation in both richness and evenness. The most diverse samples were NMF2 (Shannon = 3.43), NMS1 (Shannon = 3.30) and NMF1 (Shannon = 3.20), and the least diverse KMS1 (Shannon = 1.93), KMC1 (Shannon = 2.02) and KMC3 (Shannon = 2.04), due to the dominance of the genus *Epsilonema.* The relationship between Chao1 and Shannon was usually proportional, with the exceptions of NMC2 (Chao1 = 31 and Shannon = 2.79) and KMF1 (Chao1 = 33 and Shannon = 3.15), which had similar richness but different Shannon values, indicating that they tended to be dominated by less taxa.

The PERMANOVA main-test was significant for the factors Habitat, Site and Habitat × Site (for all, pPerm < 0.01). However, the PERMDISP analysis was not significant, indicating that the results of the PERMANOVA were not caused by heterogenic multivariate dispersion.

SIMPER analysis showed that the average similarity in group N (Senaha) was higher (52.06%) than in group K (Kouri; 41.80%), and that the main taxa responsible for this difference were *Spirophorella* sp., *Viscosia* sp. and *Croconema* sp. in group N, and Linhomoeidae, *Daptonema* sp. 1 and *Epsilonema* sp. in group K. The average dissimilarity between the two groups was 79.93% due to the higher abundance of *Viscosia* sp. and Linhomoeidae.

Focusing on habitats, the average similarity in coral samples was lower (41%) than in sand or seagrass, due to the presence of *Daptonema* sp. 1, *Spilophorella* sp. and especially *Epsilonema* sp., which was found almost only in Kouri_coral. Finally, in terms of dissimilarity, coral and seagrass showed the highest dissimilarity (81.58%), followed by sand and coral (70.82%), and finally sand and seagrass (64.99%). The main taxa determining the dissimilarity overall were *Vasostoma* sp., *Daptonema* sp. 1, *Daptonema* sp. 2, Chromadoridae, Enchelidiidae and *Molgolaimus* sp.

**Figure 4:**
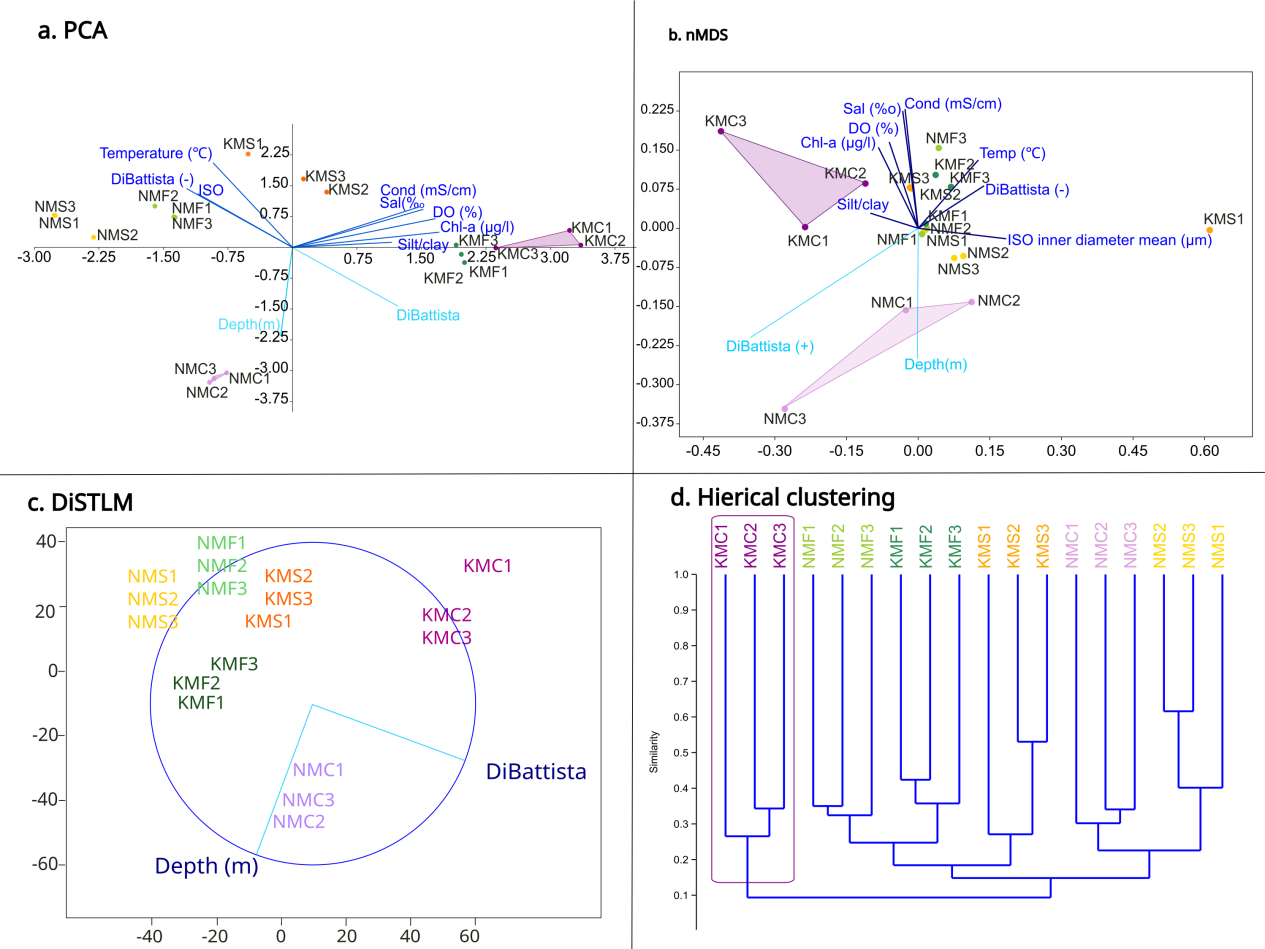
Statistical analyses based on morphological results. (A) Principal component analysis (PCA) showing the relationship between environmental variables and sites. (B) Non-metric multidimensional scaling (nMDS) showing the relationship between environmental variables, diversity and sites. (C) Distance-based multivariate multiple regression (DistLM) showing the two main parameters responsible for the variation. (D) Hieratical classic clustering of the replicates showing how samples Kouri_coral were clearly separated from the others.

The PCA (Fig. 4A) highlighted a clear separation between microhabitats mainly based on two parameters: depth and DiBattista scale. To assess the influence of these environmental variables on species composition, we also performed a DistLM analysis (Fig. 4C) marginal test (R^2^, specified), which reveals that the environmental variables of the DiBattista scale, ISO inner diameter mean, silt/clay%, temperature, salinity, conductibility, chlorophyll-a and dissolved organic were significant (*P* < 0.05). Another DistLM analysis (AICc, step-wise) also highlighted the importance of the DiBattista scale and depth as the two main factors determining the composition of nematode assemblages. The nMDS ordination (Fig. 4B) plot was elaborated including the significant environmental factors found by the DistLM. The final stress value of 0.1034 indicated the plot provided a good graphic representation of the data. The resulting plot (Fig. 4D) revealed a significantly divergent clustering of the samples in accordance with the habitat type, and coral reefs clustering separately from the others. PCA and nMDS showed similar orientations, especially for the coral reef samples from both sites.

### Molecular results and phylogeny

#### 18S rDNA

The 18S rDNA alignment consisted of 243 sequences of 767 base pairs, including 66 nematode and 2 non-nematode sequences that were retrieved from NCBI as references (S1), and 175 newly obtained sequences from this research. After selecting haplotypes with DNASP6 (S2), we obtained 104 haplotype sequences for the marker 18S which were deposited in GenBank under accession numbers: PP859916–PP859943, PP859945–PP859974, PP859976–PP859979, PP859981–PP859994, PP859996–PP860023 (S2). Among these, five sequences corresponded to taxa non previously reported in NCBI (1 x *Anonchus* Cobb, 1913; 1 x *Filoncholaimus* Filipjev, 1927; 1 x *Nannolaimoides* Ott, 1972; 2 x *Parapinnanema* Inglis, 1969 (accession numbers: PP859916, PP860023, PP859969, PP859948 and PP859949 respectively). The sequences used for the final alignment correspond to 49 taxa (1 suborder, 11 families, and 37 genera): *Acanthopharynx* (3), *Anonchus* (1), *Anticoma* (2), *Aponema* (1), Axonolaimidae (1), *Axonolaimus* (2), *Catanema* (2), *Cephalanticoma* (3), *Cheironchus* (1), *Chromadorella* (2), Chromadoridae (1), *Cobbia* (1), *Cyatholaimus* (3), *Daptonema* (2), *Desmodora* (30), Desmodoridae (2), *Desmolaimus* (1), Enchelidiidae (1), *Enoploides* (1), *Epacanthion* (3), *Eurystomina* (1), *Filoncholaimus* (1), *Fotolaimus* (1), *Laxus* (3), Linhomoeidae (1), *Mesacanthoides* (1), *Meyersia* (34), Microlaimidae. (1), *Microlaimus* (2), Monhysterina (1), *Nannolaimoides* (1), *Neochromadora* (1), Oncholaimidae (3), *Paracanthonchus* (11), *Parapinnanema* (2), *Pareurystomina* (1), Phanodermatidae (4), *Rhabdocoma* (1), *Rhabdodemania* (1), Selachinematidae (4), *Spirinia* (1), *Steineria* (1), *Theristus* (1), Thoracostomopsidae (3), *Trischistoma* (1), *Vasostoma* (3), *Viscosia* (2), Xyalidae (5), *Zalonema* (7).

**Figure 5:**
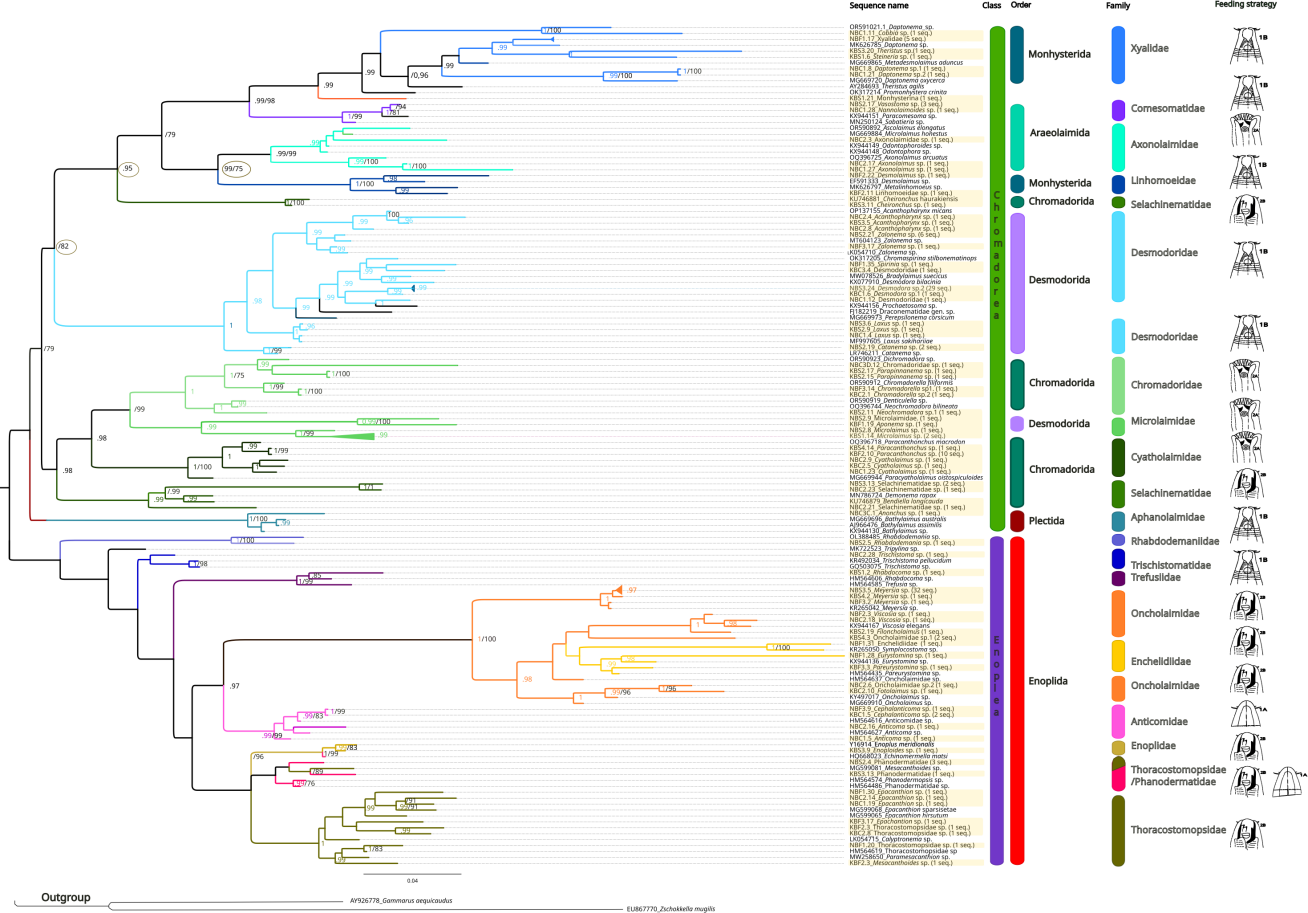
Phylogenetic tree for the 18S marker, generated by Bayesian inference. Numbers on the branches represent posterior probability and bootstrap values. below 0.97 and 75% respectively are not shown. (A) Selective deposit-feeders; (B) non-selective deposit-feeders; (A) epistrate-feeders; (B) omnivores/predators (Wieser, 1953).

The inferred phylogenetic trees exhibited similar topologies for both BI and ML analyses (Supplemental Information 3 and 4), with a clear, although not strongly supported, division between classes Chromadorea and Enoplea (Fig. 5). In total, 19 families (highlighted by different colors in Fig. 5) were recovered by the phylogenetic tree for the 18S marker generated by Bayesian Inference, of which nine were within the class Chromadorea (Xyalidae, Comesomatidae, Axonolaimidae, Linhomoeidae, Selachinematidae, Desmodoridae, Chormadoridae, Microlaimidae and Cyatholaimidae), and 10 within Enoplea (Aphanolaimidae, Rhabdodemaniidae, Trischistomatidae, Trefusiidae, Oncholaimidae, Enchelidiidae, Anticomidae, Enoplidae, Thoracostomopsidae and Phanodermatidae). Within the class Chromadorea, the clade formed by the orders Araeoloimidae+Monhysterida was supported as a separate group, similarly for the clade Chromadorida+Desmodorida (to the exception of *Cheironchus* sp., which was placed between the families Linhomoeidae and Desmodoridae). All the Chromadorea families were supported by at least one of the two analytical methods with the exception of Selachinematidae, which was split into two in both BI and ML analyses (Fig. 5, and Supplemental Information 3 and 4).

In the class Enoplea, only families Anticomidae and Enoplidae were recovered as well supported groups (posterior bootstrap, *b* = 0.97; probability, pp = 96% respectively; Fig. 5), while families Oncholaimidae and Enchelidiidae were placed into the same group. The families Oncholaimidae and Enchelidiidae appeared to be in the same clade and they were supported by both BI (value = 1) and ML (percentage = 100%).

### 28S rDNA

The 28S rDNA alignment consisted of 82 sequences of 720 base pairs, including 18 sequences that were retrieved from NCBI as references (Supplemental Information 1) and 64 new nematode sequences. After selecting haplotypes with DNASP6 (Supplemental Information 2), we obtained 37 sequences, none of which had been previously reported in NCBI (accession numbers: PP859885–PP859915). Of these, 32 were selected by the Bayesian Inference (BI) analysis to generate the 28S final tree. The number of sequences for each taxon corresponds to the following: *Anticoma* (1), *Anonchus* (1), Axonolaimidae (1), *Cephalanticoma* (1), Chromadoridae (1), *Chromadorita* (5), *Cobbia* (1), *Daptonema* (2), *Demonema* (1), *Desmodora* (11), Desmodoridae (3), Desmodoroidea (1), *Desmolaimus* (1), *Enoploides* (1), *Epacanthion* (2), *Eurystomina* (1), *Meyersia* (14), Monhysterina (1), Oncholaimidae (1), *Paracanthonchus* (5), Phanodermatidae (5), *Rhabdodemania* (1), *Viscosia* (1), *Zalonema* (2), which includes 1 suborder, 1 superfamily, 5 families, and 17 genera.

**Figure 6:**
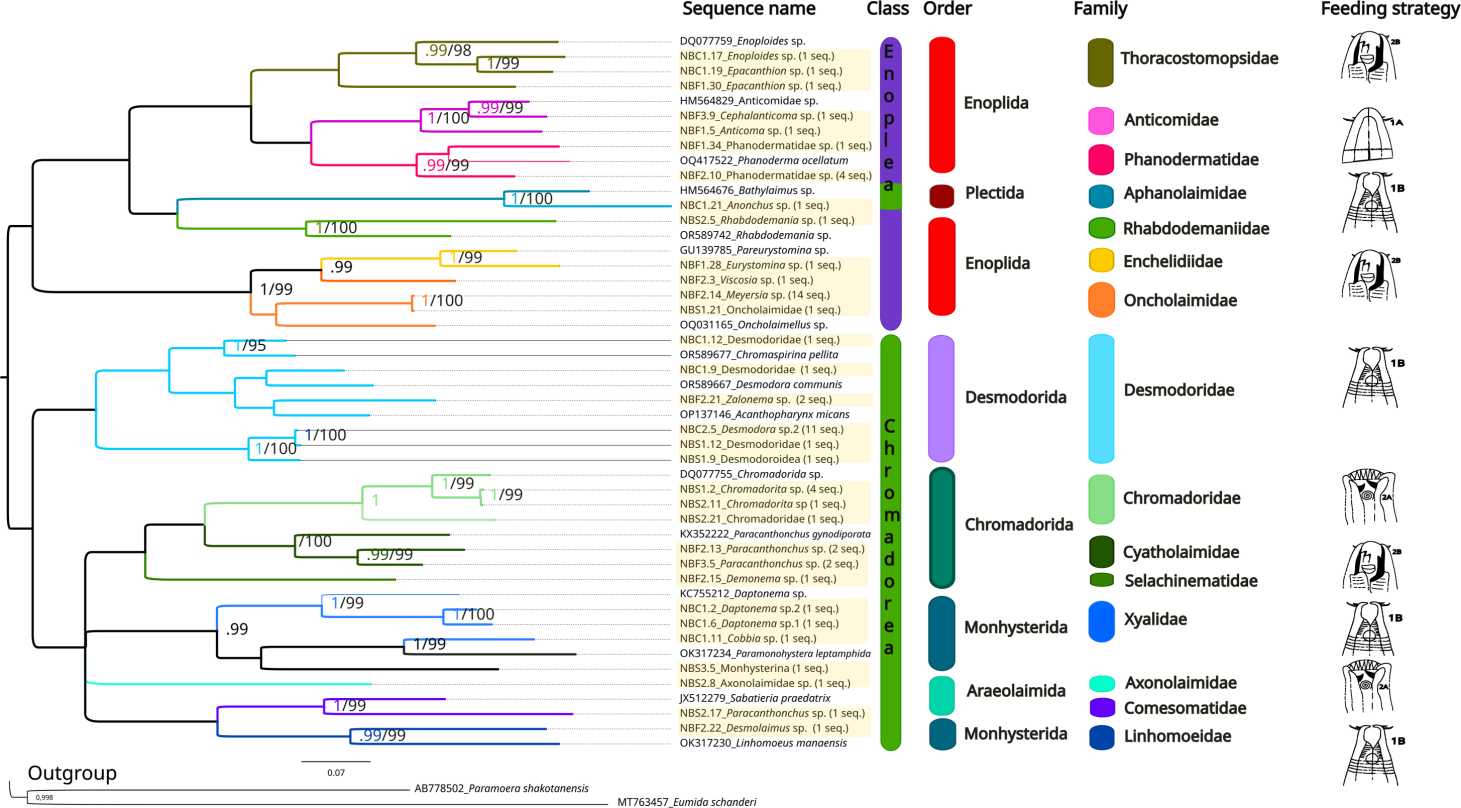
Phylogenetic tree for the 28S marker, generated by Bayesian Inference. Numbers on the branches represent posterior probability and bootstrap values. Values below 0.97 and 75% respectively are not shown. 1A, selective deposit-feeders; 1B, non-selective deposit-feeders; 2A, epistrate-feeders; 2B, omnivores/predators (Wieser, 1953).

The same methods (BI and ML) were used to reconstruct the trees for the 28S marker (Fig. 6, Supplemental Information 5 and 6), and the topologies also appeared to be similar in both analytical approaches. For 28S rDNA, eight families were found as part of the class Chromadorea (Desmodoridae, Chromadoridae, Cyatholaimidae, Selachinematidae, Xyalidae, Axonolaimidae, Comesomatidae and Linhomoeidae, but not Microlaimidae) and six in the class Enoplea (Thoracostomopsidae, Phanodermatidae, Aphanolaimidae, Rhabdodemaniidae, Enchelidiidae and Oncholaimidae, but not Trischistomatidae, Trefusiidae, Anticomidae or Enoplidae). Only the families Xyalidae, Enchelidiidae and Oncolaimidae formed well-supported clades by both BI (*pp* = 1) and ML (*b* =99%).

### Comparison of methods

**Figure 7:**
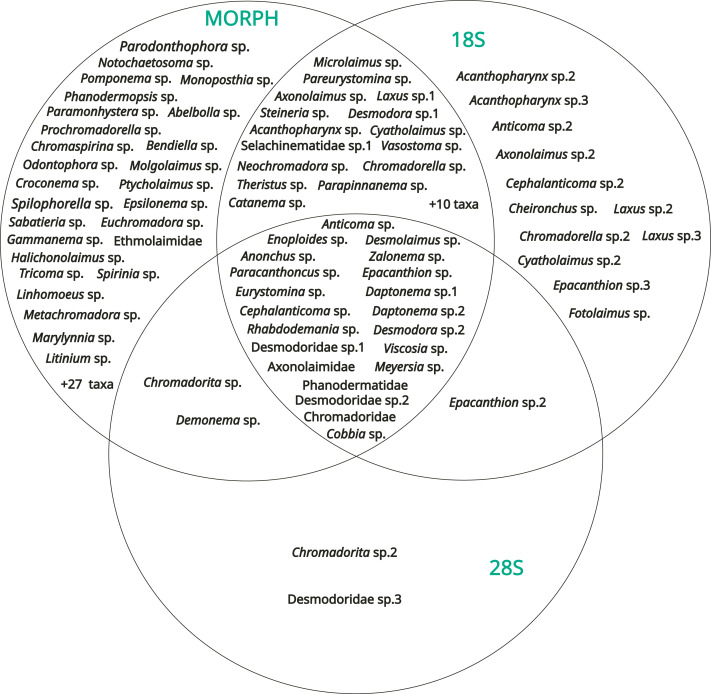
Venn diagram showing the results of the identification obtained with the three methods (morphology, DNA barcoding 18S, DNA barcoding 28S).

The results obtained from the three methods (morphology, 18S rDNA barcoding, 28S rDNA barcoding) were compared with a Venn diagram to show the overlap in taxa identification between the different methods (Fig. 7).

54 operational taxonomic units (OTUs) were identified only by morphology, including three genera determined from the SIMPER analysis [*Spilophorella* sp. (Chromadoridae), *Molgolaimus* sp. (Desmodoridae), and *Epsilonema* sp. (Epsilonematidae)]. On the other hand, 12 OTUs were amplified only by the 18S rDNA marker, two only by the 28S rDNA marker, 25 by morphology + 18S rDNA, two by morphology + 28S rDNA and one by 18S rDNA + 28S rDNA. Among the diversity of these, *Acanthopharynx* sp. (Desmodoridae), *Laxu*s sp. (Desmodoridae), and the family Thoracostomopsidae was underestimated by morphological methods. Only 21 OTUs were identified or amplified by the three methods overall, and most of the corresponding sequences of both markers did not have high levels of similarity (*e.g.*, >95%) to sequences already deposited in GenBank.

## Discussion

This study aimed to obtain a working framework of the diversity of the phylum Nematoda in two coastal coral reef sites on the west coast of Okinawa-jima Island (Senaha coast and Kouri-jima) across three habitats (sand, coral reef, seagrass beds) in order to investigate the spatial distribution of nematodes. We used different methods, a “classic” morphological taxonomic approach, and DNA barcoding of two markers, and compared the results. Our resulting dataset showed that the shallow water nematode assemblages recovered from intertidal coral reef areas of Okinawa-jima Island comprise a taxonomically diverse range of genera and families.

### Integration of morphology and DNA barcoding

The taxonomy of nematodes has always relied on morphology (Nisa, Tantray & Shah, 2022). Our molecular analyses provided partial support for the morphological identification of the free-living nematodes in this study. Overall, all methods recovered the same main families (Chromadoridae, Desmodoridae, Cyatholaimidae, Linhomoeidae and Oncholaimidae). However, discrepancies arose with the less common taxa, and morphological analyses revealed greater diversity than molecular results. Similar results have been obtained in other studies that reported higher diversity through morphology rather than molecular methods, including Abad et al. (2016) from plankton specimens of estuarine water, Dell’Anno et al. (2015) in a deep-sea study, Harvey et al. (2017) from zooplankton samples, Schenk, Kleinbölting & Traunspurger (2020) from nematodes, and Stefanni et al. (2018) from copepods. We hypothesize that low amplification success in some specimens may be due to the prevalence of small individuals, such as juveniles, or due to the small sizes of individuals of families like Desmoscolecidae and Epsilonematidae. Additionally, high nucleotide variability and indels at primer sites (Creer et al., 2010), a documented issue in free-living nematodes (Bhadury et al., 2006; De Ley et al., 2005), could have contributed to amplification failure in certain groups. The discrepancies between methods may also arise, at least in part, from the overestimation of congeneric specimens caused by the morphological plasticity typical of families such as Xyalidae and Chromadoridae. Conversely, some families, including Desmodoridae and Thoracostomopsidae, exhibit meristic features that are difficult to distinguish morphologically. For these groups, molecular methods, particularly 18S rDNA analysis, proved to be more effective. Finally, discrepancies between our morphological identifications and the closest matches in available molecular databases are most likely the result of the limited taxonomic coverage of the sequences available in GenBank relative to the vast diversity of marine nematodes (Lambshead & Boucher, 2003; Pereira et al., 2010), and the lack of a reference database from Okinawa-jima Island. Considering the sometimes-insufficient number of reference species and genera sequences deposited in GenBank (Charrier et al., 2024; Holovachov, Camp & Nadler, 2015), amplifying the available dataset of 18S and 28S genes (as they are widely used markers for invertebrates phylogenetics; (Holovachov, Camp & Nadler, 2015; Mallatt, Garey & Shultz, 2004) is essential to improve the understanding of nematode phylogeny and evolutionary history (Charrier et al., 2024). A more complete dataset (Charrier et al., 2024; Holovachov, Camp & Nadler, 2015) would improve the taxonomic coverage of underrepresented taxa or regions, reducing, in some cases, misidentifications and identification at high taxa, and speeding up the process of describing and integrating novel species in the phylogenetic tree by reducing stochastic errors (Holovachov, Camp & Nadler, 2015; Mallatt, Garey & Shultz, 2004) and making alignments more robust and better supported (Holovachov, Camp & Nadler, 2015; Mallatt, Garey & Shultz, 2004; Subbotin et al., 2008).

### Diversity and microhabitats

There is a strong correlation between environmental conditions and the composition of nematode assemblages in coral reef areas (Ruiz-Abierno & Armenteros, 2016). Among the most relevant local drivers granulometry (Gourbault & Renaud-Mornant, 1990; Heip, Vincx & Vranken, 1985; Kotta & Boucher, 2001; Netto, Warwick & Attrill, 1999; Semprucci et al., 2011), pH levels (Esteves et al., 2022), hydrodynamic conditions (Semprucci et al., 2018), periodic oxygen depletion (Boucher & Gourbault, 1990; Gourbault & Renaud-Mornant, 1990; Kotta & Boucher, 2001; Sournia, 1976), depth (Netto, Warwick & Attrill, 1999), and the presence of coral fragments (Netto, Warwick & Attrill, 1999; Raes, Decraemer & Vanreusel, 2008) have been mentioned in the literature.

In our study, we observed a positive correlation between the Shannon (which represents richness and evenness) and Chao1 (which represents richness) indices. This suggests that communities with a higher number of taxa also tended to exhibit greater evenness (Konopiński, 2020; Morris et al., 2014; Voutilainen & Kangasniemi, 2015). Samples from Senaha Beach generally had both higher Chao1 and Shannon compared to Kouri, except for KMF1 (Kouri_seagrass 1). While the seagrass habitats had higher diversity and richness, followed by Senaha_coral, sand habitats and Kouri_coral, our morphological results showed that coral reef habitats were different from sand and seagrass beds in terms of nematode generic assemblages. Coral reef habitats stood out in terms of dissimilarity from both sand and seagrass beds, and in particular Kouri_coral, while having low diversity compared to the other microhabitats, although they had unique nematode assemblages mainly due to the presence of *Epsilonema*, *Spilophorella*, Enchelidiidae, *Daptonema*, *Molgolaimus*, *Viscosia*, Linhomoeidae, and *Vasostoma*). In addition, PCA analyses and nMDS ordination clearly showed coral sites as different from the other sites in terms of environmental parameters and diversity, with the main two factors being depth (an artifact of our sampling design) and anthropogenic pressures/impacts. Even considering that this research was limited to only two sites, both coral habitats showed the lowest similarity among themselves and the highest dissimilarity from the other habitats. These results suggest that microhabitats can be a potential driver in structuring nematode diversity at least in our examined sites.

Morphological evidence from other parts of the world (Armenteros et al., 2009; Armenteros & Ruiz-Abierno, 2015; Semprucci et al., 2013; Semprucci et al., 2018) has revealed that the main genera appear to be ubiquitous worldwide. For example, the genus *Epsilonema* was also found in coral reef fragments from the Porcupine Seabight (NE Atlantic) by Raes & Vanreusel (2006) and it was one of three most common genera found in Kenya and Zanzibar by Raes et al. (2007). *Spilophorella* and *Viscosia* were two of the top three genera discovered in one site of a study conducted in Taiwan by Wei-Ling et al. (2022), and *Spilophorella* is described as having the potential to be used as an indicator for a healthy environment (Wei-Ling et al., 2022). *Spilophorella* was also recorded as a common genus, for example, in the central Great Barrier Reef (Tietjen, 1991) and in the Caribbean (Ruiz-Abierno & Armenteros, 2016) and in Shimoni (Kenya) by Hashim et al. (2022). The genus *Daptonema* is widely distributed in diverse substrates globally, including coral reefs (Wei-Ling et al., 2022; Grassi et al., 2022; Semprucci et al., 2013). *Viscosia* is another common and ubiquitous genus, and in a few sites was recorded as the only genus present (Mohammad, 2022; Samad et al., 2018) or as part of non-diverse communities (Sherman, 1985). *Molgolaimus* has been recorded from mangrove areas (*e.g.*, Sun & Huang, 2024; Zhou et al., 2020), but not directly associated with coral reefs. Finally, there are no direct records of *Vasostoma* in coral reef areas, but the genus was found in three Indian harbors (Nanajkar & Ingole, 2010) and from the continental slope in New Zealand (Leduc & Nodder, 2012).

However, the most frequent taxa are generally ubiquitous, while numerous novel genera and species have been recently discovered in tropical areas including the East China Sea (Chen & Guo, 2014; Chen & Guo, 2015; Chunming, Liguo & Yong, 2015; Leduc, 2022; Leduc & Sinniger, 2018; Lu, Sui & Huang, 2022; Semprucci & Balsamo, 2014; Sun, Huang & Huang, 2018; Sun, Huang & Huang, 2021). Our results strongly suggest that it is worth investigating nematode communities in coral reefs much more closely in Okinawa-jima Island as they may be a possible hotspot of diversity.

### Topology of the phylum Nematoda

Regarding the structure of the phylogenetic trees we recovered in this study, less OTUs were recovered by 28S rDNA when compared to 18S rDNA, but the two markers (18S rDNA, 28S rDNA) and both ML and Bayesian trees showed similar topologies (Figs. 4 and 5), in accordance with previous studies (Supplemental Information 8; Armenteros et al., 2014; Avó et al., 2017; Bik et al., 2010a; Bik et al., 2010b; Derycke et al., 2010; Kumar, Sen & Bhadury, 2014; Pereira et al., 2010). Our recovered general topology was coherent with those in earlier findings (Ahmed, Boström & Holovachov, 2020; Smythe, Holovachov & Kocot, 2019), although the orders Enoplea and Chromadorea, recovered in all analyses, were not well supported.

18S rDNA proved to be more efficient than 28S rDNA in obtaining good quality sequences and resolving nematode phylogeny at the family level. The 18S rDNA results highlighted the monophyly of the families Xyalidae, Comesomatidae, Axonolaimidae, Linhomoeidae and Chromadoridae. It should be noted that the monophyly of the family Desmodoridae is controversial, and in accordance with Armenteros et al. (2014), Kumar, Sen & Bhadury (2014) and Meldal et al. (2007), our ML analysis supported this group, while the Bayesian analysis did not, with Epsilonematidae and Draconematidae sequences placed in the same clade. Inferring relationships within the Desmodoroidea is limited by the often fragmentary and potentially erroneous nature of the available molecular data and this situation may be responsible for the unrealistic genetic distances observed within some species and genera (Leduc & Zhao, 2021). Within the family Desmodoridae, phylogenetic relationships among the genera were partly resolved. Our study supported the monophyly of the genera *Acanthopharynx*, *Zalonema*, *Catanema* and *Laxus*, in contradiction with Armenteros et al. (2014), but not the genus *Desmodora,* for which the phylogenetic position remained ambiguous, possibly due to misidentifications in past research. Based on the World Register of Marine Species (WoRMS Editorial Board, 2024), *Desmodora* is the most diverse genus of the family Desmodoridae, currently containing 70 valid species, and many changes have been made within the genus as the identification of members this genus is challenging due to a limited number of useful differential morphological characters (Mordukhovich et al., 2023).

In accordance with Bik et al. (2010b) and Pereira et al. (2010), our analyses also supported the hypothesis that Oncholaimidae and Enchelidiidae may not be two different families, as our 18S and 28S rDNA results placed them in the same clade. In addition, we observed that they were very similar morphologically, particularly regarding the shape of the mouth and their sizes.

## Conclusions

As this is one of the first attempts to focus on nematode assemblages in the shallow marine waters of Okinawa-jima Island, we could not directly compare our overall diversity data with previous studies, but our results suggest that nematode diversity in Okinawa is overwhelmingly high it is currently difficult to accurately determine due to the lack of baseline data, which this research provides. We identified at least 10 orders and 38 genera-level groups of nematodes from our samples that only span two different sites, and it is highly likely these samples include undescribed taxa. As other research has suggested, we demonstrated that morphological and DNA barcoding methods tended to reveal somewhat different results, particularly for less prevalent nematode taxa, perhaps due to morphological misidentifications stemming from ontogenetic features and morphological plasticity, and also likely due to a lack of sequences deposited in GenBank. For this reason, an integrated approach is recommended when it comes to studying the diversity of nematodes in areas not yet known, such as Okinawa and the Ryukyus (Reimer et al., 2019).

For instance, the phylogenetic analysis revealed that most genera within the family Desmodoridae are monophyletic, while the genus *Desmodora* appears to be more complex and potentially polyphyletic. Finally, the structure of the trees we obtained offers insights into the topology of the phylum Nematoda, suggesting that Oncholaimidae and Enchelidiidae might not be two different families. Overall, our study showed Okinawan coral reefs and neighboring areas are potential hot-spots of diversity for free-living marine nematodes, and that nematode communities here consist of a taxonomically diverse group of genera and families. The data acquired from this study represents the start of a baseline reference database for nematodes in Okinawa-jima Island, and it is hoped that future studies will build on our current findings.

## Supplemental Information

10.7717/peerj.19757/supp-1 Supplemental Information 1 List of NCBI sequences used as reference to build the phylogenetic trees

10.7717/peerj.19757/supp-2 Supplemental Information 2 List of haplotypes used to build the phylogenetic trees

10.7717/peerj.19757/supp-3 Supplemental Information 3 Tree 18S_bayes only

10.7717/peerj.19757/supp-4 Supplemental Information 4 Tree 18S ML only

10.7717/peerj.19757/supp-5 Supplemental Information 5 Tree 28S bayes only

10.7717/peerj.19757/supp-6 Supplemental Information 6 Tree 28S ML only

10.7717/peerj.19757/supp-7 Supplemental Information 7 List of nematode taxa obtained through morphology

10.7717/peerj.19757/supp-8 Supplemental Information 8 Table of papers related to molecular work on nematodes

## Additional Information and Declarations

### DNA Deposition

The following information was supplied regarding the deposition of DNA sequences: The newly obtained DNA sequences are available at GenBank: PP859885–PP859943, PP859945–PP859974, PP859976–PP859979, PP859981–PP859994, PP859996–PP860023.

### Data Availability

The following information was supplied regarding data availability: The raw data are available in the Supplementary Files.

## References

[ref-1] Abad D, Albaina A, Aguirre M, Laza-Martínez A, Uriarte I, Iriarte A, Villate F, Estonba A (2016). Is metabarcoding suitable for estuarine plankton monitoring? A comparative study with microscopy. Marine Biology.

[ref-2] Ahmed M, Boström S, Holovachov O (2020). Revision of the genus *Cobbionema* Filipjev, 1922 (Nematoda, Chromadorida, Selachinematidae). European Journal of Taxonomy.

[ref-3] Altschul SF, Gish W, Miller W, Myers EW, Lipman DJ (1990). Basic local alignment search tool. Journal of Molecular Biology.

[ref-4] Anderson MJ (2001). A new method for non-parametric multivariate analysis of variance. Austral Ecology.

[ref-5] Appeltans W, Ahyong ST, Anderson G, Angel MV, Artois T, Bailly N, Bamber R, Barber A, Bartsch I, Berta A, Błażewicz Paszkowycz M, Bock P, Boxshall G, Boyko CB, Brandão SN, Bray RA, Bruce NL, Cairns SD, Chan T-Y, Cheng L, Collins AG, Cribb T, Curini-Galletti M, Dahdouh-Guebas F, Davie PJF, Dawson MN, De Clerck O, Decock W, De Grave S, De Voogd NJ, Domning DP, Emig CC, Erséus C, Eschmeyer W, Fauchald K, Fautin DG, Feist SW, Fransen CHJM, Furuya H, Garcia-Alvarez O, Gerken S, Gibson D, Gittenberger A, Gofas S, Gómez-Daglio L, Gordon DP, Guiry MD, Hernandez F, Hoeksema BW, Hopcroft RR, Jaume D, Kirk P, Koedam N, Koenemann S, Kolb JB, Kristensen RM, Kroh A, Lambert G, Lazarus DB, Lemaitre R, Longshaw M, Lowry J, Macpherson E, Madin LP, Mah C, Mapstone G, McLaughlin PA, Mees J, Meland K, Messing CG, Mills CE, Molodtsova TN, Mooi R, Neuhaus B, Ng PKL, Nielsen C, Norenburg J, Opresko DM, Osawa M, Paulay G, Perrin W, Pilger JF, Poore GCB, Pugh P, Read GB, Reimer JD, Rius M, Rocha RM, Saiz-Salinas JI, Scarabino V, Schierwater B, Schmidt-Rhaesa A, Schnabel KE, Schotte M, Schuchert P, Schwabe E, Segers H, Self-Sullivan C, Shenkar N, Siegel V, Sterrer W, Stöhr S, Swalla B, Tasker ML, Thuesen EV, Timm T, Antonio Todaro M, Turon X, Tyler S, Uetz P, Van der Land J, Vanhoorne B, Van Ofwegen LP, Van Soest RWM, Vanaverbeke J, Walker-Smith G, Chad Walter T, Warren A, Williams GC, Wilson SP, Costello MJ (2012). The magnitude of global marine species diversity. Current Biology.

[ref-6] Armenteros M, Rojas-Corzo A, Ruiz-Abierno A, Derycke S, Backeljau T, Decraemer W (2014). Systematics and DNA barcoding of free-living marine nematodes with emphasis on tropical desmodorids using nuclear SSU rDNA and mitochondrial COI sequences. Nematology.

[ref-7] Armenteros M, Ruiz-Abierno A (2015). Body size distribution of free-living marine nematodes from a Caribbean coral reef. Nematology.

[ref-8] Armenteros M, Ruiz-Abierno A, Fernández-Garcés R, Pérez-García JA, Díaz-Asencio L, Vincx M, Decraemer W (2009). Biodiversity patterns of free-living marine nematodes in a tropical bay: Cienfuegos, Caribbean Sea. Estuarine, Coastal and Shelf Science.

[ref-9] Avó AP, Daniell TJ, Neilson R, Oliveira S, Branco J, Adão H (2017). DNA barcoding and morphological identification of benthic nematodes assemblages of estuarine intertidal sediments: advances in molecular tools for biodiversity assessment. Frontiers in Marine Science.

[ref-10] Bhadury P, Austen M, Bilton D, Lambshead P, Rogers A, Smerdon G (2006). Development and evaluation of a DNA-barcoding approach for the rapid identification of nematodes. Marine Ecology Progress Series.

[ref-11] Bik HM, Lambshead PJD, Thomas WK, Lunt DH (2010b). Moving towards a complete molecular framework of the Nematoda: a focus on the Enoplida and early-branching clades. BMC Evolutionary Biology.

[ref-12] Bik HM, Thomas WK, Lunt DH, Lambshead PJD (2010a). Low endemism, continued deep-shallow interchanges, and evidence for cosmopolitan distributions in free-living marine nematodes (order Enoplida). BMC Evolutionary Biology.

[ref-13] Blaxter ML, De Ley P, Garey JR, Liu LX, Scheldeman P, Vierstraete A, Vanfleteren JR, Mackey LY, Dorris M, Frisse LM, Vida JT, Thomas WK (1998). A molecular evolutionary framework for the phylum Nematoda. Nature.

[ref-14] Blott SJ, Pye K (2012). Particle size scales and classification of sediment types based on particle size distributions: review and recommended procedures. Sedimentology.

[ref-15] Blouin MS, Yowell CA, Courtney CH, Dame JB (1998). Substitution bias, rapid saturation, and the use of mtDNA for nematode systematics. Molecular Biology and Evolution.

[ref-16] Bogale M, Baniya A, Di Gennaro P (2020). Nematode identification techniques and recent advances. Plants.

[ref-17] Boucher G, Gourbault N (1990). Sublittoral meiofauna and diversity of nematode assemblages off Guadeloupe Islands (French West Indies). Bulletin of Marine Science.

[ref-18] Boufahja F, Hedfi A, Amorri J, Aïssa P, Beyrem H, Mahmoudi E (2010). An assessment of the impact of chromium-amended sediment on a marine nematode assemblage using microcosm bioassays. Biological Trace Element Research.

[ref-19] Burgess R (2001). An improved protocol for separating meiofauna from sediments using colloidal silica sols. Marine Ecology Progress Series.

[ref-20] Chao A (1984). Non-parametric estimation of the number of classes in a population. Scandinavian Journal of Statistics.

[ref-21] Chariton AA, Ho KT, Proestou D, Bik H, Simpson SL, Portis LM, Cantwell MG, Baguley JG, Burgess RM, Pelletier MM, Perron M, Gunsch C, Matthews RA (2014). A molecular-based approach for examining responses of eukaryotes in microcosms to contaminant-spiked estuarine sediments. Environmental Toxicology and Chemistry.

[ref-22] Charrier E, Chen R, Thundathil N, Gilleard JS (2024). A set of nematode rRNA cistron databases and a primer assessment tool to enable more flexible and comprehensive metabarcoding. Molecular Ecology Resources, Early View.

[ref-23] Chen YZ, Guo YQ (2014). Three new species of free-living marine nematodes from East China Sea. Zootaxa.

[ref-24] Chen YZ, Guo YQ (2015). Three new and two known free-living marine nematode species of the family Ironidae from the East China Sea. Zootaxa.

[ref-25] Chunming W, Liguo A, Yong H (2015). A new species of free-living marine nematode (Nematoda: Chromadoridae) from the East China Sea. Zootaxa.

[ref-26] Creer S, Fonseca VG, Porazinska DL, Giblin-Davis RM, Sung W, Power DM, Packer M, Carvalho GR, Blaxter ML, Lambshead PJD, Thomas WK (2010). Ultrasequencing of the meiofaunal biosphere: practice, pitfalls and promises. Molecular Ecology.

[ref-27] De Ley P (2006). A quick tour of nematode diversity and the backbone of nematode phylogeny. WormBook, The C. elegans Research Community, ed. WormBook.

[ref-28] De Ley P, De Ley IT, Morris K, Abebe E, Mundo-Ocampo M, Yoder M, Heras J, Waumann D, Rocha-Olivares A, Jay Burr AH, Baldwin JG, Thomas WK (2005). An integrated approach to fast and informative morphological vouchering of nematodes for applications in molecular barcoding. Philosophical Transactions of the Royal Society B: Biological Sciences.

[ref-29] Dell’Anno A, Carugati L, Corinaldesi C, Riccioni G, Danovaro R (2015). Unveiling the biodiversity of deep-sea nematodes through metabarcoding: are we ready to bypass the classical taxonomy?. PLOS ONE.

[ref-30] Derycke S, Remerie T, Vierstraete A, Backeljau T, Vanfleteren J, Vincx M, Moens T (2005). Mitochondrial DNA variation and cryptic speciation within the free-living marine nematode *Pellioditis marina*. Marine Ecology Progress Series.

[ref-31] Derycke S, Vanaverbeke J, Rigaux A, Backeljau T, Moens T (2010). Exploring the use of Cytochrome Oxidase c Subunit 1 (COI) for DNA barcoding of free-living marine nematodes. PLOS ONE.

[ref-32] DiBattista JD, Reimer JD, Stat M, Masucci GD, Biondi P, De Brauwer M, Wilkinson SP, Chariton AA, Bunce M (2020). Environmental DNA can act as a biodiversity barometer of anthropogenic pressures in coastal ecosystems. Scientific Reports.

[ref-33] Edgar RC (2004). MUSCLE: multiple sequence alignment with high accuracy and high throughput. Nucleic Acids Research.

[ref-34] Esteves AM, Souza TP, Da Sarmento VC, Maria TF, Dos Santos PJP (2022). Effects of the ocean acidification on the functional structure of coral reef nematodes. Coral Reefs.

[ref-35] Fadeeva N, Mordukhovich V, Zograf J (2016). Free-living marine nematodes of *Desmodorella* and *Zalonema* (Nematoda: Desmodoridae) with description of two new species from the deep sea of the North Western Pacific. Zootaxa.

[ref-36] Gao C (2023). https://cran.r-project.org/package=ggVennDiagram.

[ref-37] Gourbault N, Renaud-Mornant J (1990). Micro-Meiofaunal community structure and nematode diversity in a lagoonal ecosystem (Fangataufa, eastern Tuamotu archipelago). Marine Ecology.

[ref-38] Grassi E, Montefalcone M, Cesaroni L, Guidi L, Balsamo M, Semprucci F (2022). Taxonomic and functional nematode diversity in Maldivian coral degradation zones: patterns across reef typologies and depths. PeerJ.

[ref-39] Haendiges J, Timme R, Kastanis G, Balkey M (2020). Manual DNA extraction using qiagen DNeasy blood and tissue kit v1.

[ref-40] Hammer O, Harper DAT, Ryan PD (2001). PAST: Paleontological statistics software package for education and data analysis. Palaeontologia Electronica.

[ref-41] Harvey JB, Johnson SB, Fisher JL, Peterson WT, Vrijenhoek RC (2017). Comparison of morphological and next generation DNA sequencing methods for assessing zooplankton assemblages. Journal of Experimental Marine Biology and Ecology.

[ref-42] Hashim SM, Muthumbi AWN, Githaiga JM, Okondo J (2022). Nematode community structure and distribution along the Kenyan continental shelf. African Journal of Marine Science.

[ref-43] Heip CHR, Vincx M, Vranken G (1985). The ecology of marine nematodes. Oceanography and Marine Biology: An Annual Review.

[ref-44] Holovachov O, Camp L, Nadler SA (2015). Sensitivity of ribosomal RNA character sampling in the phylogeny of Rhabditida. Journal of Nematology.

[ref-45] Inkscape Project (2020). https://inkscape.org.

[ref-46] Kearse M, Moir R, Wilson A, Stones-Havas S, Cheung M, Sturrock S, Buxton S, Cooper A, Markowitz S, Duran C, Thierer T, Ashton B, Meintjes P, Drummond A (2012). Geneious basic: an integrated and extendable desktop software platform for the organization and analysis of sequence data. v2023.2 (Version 2032.3). Bioinformatics.

[ref-47] Konopiński MK (2020). Shannon diversity index: a call to replace the original Shannon’s formula with unbiased estimator in the population genetics studies. PeerJ.

[ref-48] Kotta J, Boucher G (2001). Interregional variation of free-living nematode assemblages in tropical coral sands. Cahiers de Biologie Marine.

[ref-49] Kumar A, Sen D, Bhadury P (2014). Unraveling free-living marine nematode community structure from a biodiversity-rich tropical coastal setting based on molecular approaches. Marine Biodiversity.

[ref-50] Lambshead PJD (2004). Marine nematode biodiversity. Nematology: advances and perspectives. Volume 1: nematode morphology, physiology, and ecology.

[ref-51] Lambshead PJD, Boucher G (2003). Marine nematode deep-sea biodiversity—hyperdiverse or hype?. Journal of Biogeography.

[ref-52] Leduc D (2013). Two new genera and five new species of Selachinematidae (Nematoda, Chromadorida) from the continental slope of New Zealand. European Journal of Taxonomy.

[ref-53] Leduc D (2022). Zalonema sesokoensis n. sp. (Nematoda: Desmodoridae), a new nematode species from subtidal sediments of Sesoko Island, Japan. Galaxea, Journal of Coral Reef Studies.

[ref-54] Leduc D, Nodder SD (2012). Unravelling the environmental drivers of deep-sea nematode diversity on the New Zealand continental margin. Deep-Sea Research Part I: Oceanographic Research Papers.

[ref-55] Leduc D, Sinniger F (2018). Combining morphological and molecular data to classify *Laxus sakihariiae* sp. n. a new stilbonematine nematode (Nematoda: Desmodoridae) from the coast of Sesoko Island, Japan. Nematology.

[ref-56] Leduc D, Zhao ZQ (2021). Molecular characterization of free-living nematodes from Kermadec Trench (Nematoda: Aegialoalaimidae, Xyalidae) with description of *Aegialoalaimus tereticauda* n. sp. Zootaxa.

[ref-57] Litvaitis MK, Bates JW, Hope WD, Moens T (2000). Inferring a classification of the Adenophorea (Nematoda) from nucleotide sequences of the D3 expansion segment (26/28S rDNA). Canadian Journal of Zoology.

[ref-58] Lu Y, Sui X, Huang Y (2022). Three new species of free-living marine nematodes from the central basin of the South China Sea. Journal of Natural History.

[ref-59] Mallatt JM, Garey JR, Shultz JW (2004). Ecdysozoan phylogeny and Bayesian inference: first use of nearly complete 28S and 18S rRNA gene sequences to classify the arthropods and their kin. Molecular Phylogenetics and Evolution.

[ref-60] Masucci GD, Reimer JD (2019). Expanding walls and shrinking beaches: loss of natural coastline in Okinawa Island, Japan. PeerJ.

[ref-61] Meiri S (2018). The smartphone fallacy—when spatial data are reported at spatial scales finer than the organisms themselves. Frontiers of Biogeography.

[ref-62] Meldal BHM, Debenham NJ, De Ley P, De Ley IT, Vanfleteren JR, Vierstraete AR, Bert W, Borgonie G, Moens T, Tyler PA, Austen MC, Blaxter ML, Rogers AD, Lambshead PJD (2007). An improved molecular phylogeny of the Nematoda with special emphasis on marine taxa. Molecular Phylogenetics and Evolution.

[ref-63] Miller MA, Pfeiffer W, Schwartz T (2010). Creating the CIPRES science gateway for inference of large phylogenetic trees.

[ref-64] Moens T, Braeckman U, Derycke S, Fonseca G, Gallucci F, Gingold R, Guilini K, Ingels J, Leduc D, Vanaverbeke J, Van Colen C, Vanreusel A, Vincx M, Schmidt-Rhaesa A (2013). Ecology of free-living marine nematodes. Handbook of zoology: gastrotricha, cycloneuralia and gnathifera. Volume 2: Nematoda.

[ref-65] Mohammad DA (2022). Meiobenthic assemblages in some coral reef sites in Marsa Alam (Red Sea, Egypt) with emphasis on free living nematodes. Egyptian Journal of Aquatic Biology and Fisheries.

[ref-66] Mordukhovich VV, Fadeeva NP, Semenchenko AA, Kiyashko SI, Scripova ER (2023). New and known species of the genus *Desmodora* De Man, 1889 (Nematoda: Desmodoridae) from the hydrothermal vent communities of the Piip volcano (south-west Bering Sea). Deep Sea Research Part II: Topical Studies in Oceanography.

[ref-67] Morris EK, Caruso T, Buscot F, Fischer M, Hancock C, Maier TS, Meiners T, Müller C, Obermaier E, Prati D, Socher SA, Sonnemann I, Wäschke N, Wubet T, Rillig MC (2014). Choosing and using diversity indices: insights for ecological applications from the German biodiversity exploratories. Ecology and Evolution.

[ref-68] Muthumbi AW, Vincx M (1999). Microlaimidae (Microlaimoidea: Nematoda) from the Indian Ocean: description of nine new and known species. Hydrobiologia.

[ref-69] Nadler SA (1992). Phylogeny of some ascaridoid nematodes, inferred from comparison of 18S and 28S rRNA sequences. Molecular Biology and Evolution.

[ref-70] Nadler SA, Carreno RA, Mejía-Madrid H, Ullberg J, Pagan C, Houston R, Hugot JP (2007). Molecular phylogeny of clade III nematodes reveals multiple origins of tissue parasitism. Parasitology.

[ref-71] Nanajkar M, Ingole BS (2010). Impact of sewage disposal on a nematode community of a tropical sandy beach. Journal of Environmental Biology.

[ref-72] Nemys (eds) (2024). https://nemys.ugent.be.

[ref-73] Netto S, Warwick R, Attrill M (1999). Meiobenthic and macrobenthic community structure in carbonate sediments of Rocas Atoll (North-east, Brazil). Estuarine Coastal and Shelf Science.

[ref-74] Nisa RU, Tantray AY, Shah AA (2022). Shift from morphological to recent advanced molecular approaches for the identification of nematodes. Genomics.

[ref-75] Nunn GB (1992). Nematode molecular evolution. Ph.D. Thesis.

[ref-76] Oksanen J, Simpson GL, Blanchet FG, Kindt R, Legendre P, Minchin PR, O’Hara RB, Solymos P, Stevens MHH, Wagner H (2025). https://cran.r-project.org/package=vegan.

[ref-77] Pereira TJ, Fonseca G, Mundo-Ocampo M, Guilherme BC, Rocha-Olivares A (2010). Diversity of free-living marine nematodes (Enoplida) from Baja California assessed by integrative taxonomy. Marine Biology.

[ref-78] Platt HM, Warwick RM (1983). Free-living marine Nematodes. Part 1 British Enoplids book.

[ref-79] Platt HM, Warwick RM (1988). Free-living marine Nematodes. Part II. British Chromadorids; Synopses of the British Fauna, Volume 38.

[ref-80] QGIS Development Team (2024). http://qgis.org.

[ref-81] R Core Team (2025). https://www.R-project.org/.

[ref-82] Raes M, De Troch M, Ndaro SGM, Muthumbi A, Guilini K, Vanreusel A (2007). The structuring role of microhabitat type in coral degradation zones: a case study with marine nematodes from Kenya and Zanzibar. Coral Reefs.

[ref-83] Raes M, Decraemer W, Vanreusel A (2008). Walking with worms: coral-associated epifaunal nematodes. Journal of Biogeography.

[ref-84] Raes M, Vanreusel A (2006). Microhabitat type determines the composition of nematode communities associated with sediment-clogged cold-water coral framework in the Porcupine Seabight (NE Atlantic).

[ref-85] Rambaut A (2018). http://tree.bio.ed.ac.uk/software/figtree/.

[ref-86] Reimer JD, Biondi P, Lau YW, Masucci GD, Nguyen XH, Santos ME, Wee HB (2019). Marine biodiversity research in the Ryukyu Islands, Japan: current status and trends. PeerJ.

[ref-87] Ridall A, Ingels J (2021). Suitability of free-living marine nematodes as bioindicators: status and future considerations. Frontiers in Marine Science.

[ref-88] Roberts CM, McClean CJ, Veron JEN, Hawkins JP, Allen GR, McAllister DE, Mittermeier CG, Schueler FW, Spalding M, Wells F, Vynne C, Werner TB (2002). Marine biodiversity hotspots and conservation priorities for tropical reefs. Science.

[ref-89] Ronquist F, Teslenko M, Van der Mark P, Ayres DL, Darling A, Höhna S, Larget B, Liu L, Suchard MA, Huelsenbeck JP (2012). MrBayes 3.2: efficient bayesian phylogenetic inference and model choice across a large model space. Systematic Biology.

[ref-90] Rozas J, Ferrer-Mata A, Sánchez-DelBarrio JC, Guirao-Rico S, Librado P, Ramos-Onsins SE, Sánchez-Gracia A (2017). DnaSP 6: DNA sequence polymorphism analysis of large data sets. Molecular Biology and Evolution.

[ref-91] Ruiz-Abierno A, Armenteros M (2016). Coral reef habitats strongly influence the diversity of macro- and meiobenthos in the Caribbean. Marine Biodiversity.

[ref-92] Samad S, Mohammad M, Salleh S, Darif A (2018). A checklist of free-living marine nematodes at different ecosystem in Northern Straits of Malacca, Malaysia. Sains Malaysiana.

[ref-93] Schenk J, Kleinbölting N, Traunspurger W (2020). Comparison of morphological, DNA barcoding, and metabarcoding characterizations of freshwater nematode communities. Ecology and Evolution.

[ref-94] Seinhorst JW (1962). Modifications of the elutriation method for extracting nematodes from soil. Nematologica.

[ref-95] Semprucci F, Balsamo M (2014). New records and distribution of marine free-living nematodes in the Maldivian Archipelago. Proceedings of the Biological Society of Washington.

[ref-96] Semprucci F, Colantoni P, Baldelli G, Sbrocca C, Rocchi M, Balsamo M (2013). Meiofauna associated with coral sediments in the Maldivian subtidal habitats (Indian Ocean). Marine Biodiversity.

[ref-97] Semprucci F, Colantoni P, Sbrocca C, Baldelli G, Rocchi M, Balsamo M (2011). Meiofauna in sandy back-reef platforms differently exposed to the monsoons in the Maldives (Indian Ocean). Journal of Marine Systems.

[ref-98] Semprucci F, Frontalini F, Losi V, Armynotdu Châtelet E, Cesaroni L, Sandulli R, Coccioni R, Balsamo M (2018). Biodiversity and distribution of the meiofaunal community in the reef slopes of the Maldivian archipelago (Indian Ocean). Marine Environmental Research.

[ref-99] Shannon CE (1948). A mathematical theory of communication. Bell System Technical Journal.

[ref-100] Sherman KM (1985). Ecological investigations of the epifauna and flora of bay scallops, with special reference to free-living nematodes. Ph.D. thesis.

[ref-101] Shilla DJ, Mimura I, Takagi KK, Tsuchiya M (2013). Preliminary survey of the nutrient discharge characteristics of Okinawa Rivers, and their potential effects on inshore coral reefs. Galaxea, Journal of Coral Reef Studies.

[ref-102] Smythe AB, Holovachov O, Kocot KM (2019). Improved phylogenomic sampling of free-living nematodes enhances resolution of higher-level nematode phylogeny. BMC Evolutionary Biology.

[ref-103] Sournia A (1976). Primary production of sands in the lagoon of an atoll and the role of foraminiferan symbionts. Marine Biology.

[ref-104] Stefanni S, Stanković D, Borme D, De Olazabal A, Juretić T, Pallavicini A, Tirelli V (2018). Multi-marker metabarcoding approach to study mesozooplankton at basin scale. Scientific Reports.

[ref-105] Subbotin SA, Ragsdale EJ, Mullens T, Roberts PA, Mundo-Ocampo M, Baldwin JG (2008). A phylogenetic framework for root lesion nematodes of the genus *Pratylenchus* (Nematoda): evidence from 18S and D2–D3 expansion segments of 28S ribosomal RNA genes and morphological characters. Molecular Phylogenetics and Evolution.

[ref-106] Sun Y, Huang M, Huang Y (2018). Two new species of free-living nematodes from the East China Sea. Acta Oceanologica Sinica.

[ref-107] Sun J, Huang M, Huang Y (2021). Four new species of free-living marine nematode from the sea areas of China. Journal of Oceanology and Limnology.

[ref-108] Sun J, Huang Y (2024). *Metachromadora parobscura* sp. nov. and *Molgolaimuslongicaudatus* sp. nov. (Nematoda, Desmodoridae) from Mangrove Wetlands of China. Journal of Marine Science and Engineering.

[ref-109] Takeuchi I (2023). Anthropogenic stresses in coral reefs and adjacent ecosystems of the East China Sea. Coral Reefs of the World.

[ref-110] Tamura K, Stecher G, Kumar S (2021). MEGA11: molecular evolutionary genetics analysis v11.0.10 (Version 11). Molecular Biology and Evolution.

[ref-111] Tietjen JH (1991). Ecology of free-living nematodes from the continental shelf of the central Great Barrier Reef province. Estuarine, Coastal and Shelf Science.

[ref-112] Voutilainen A, Kangasniemi M (2015). Applying the ecological Shannon’s diversity index to measure intragroup collaboration diversity. Journal of Scientometric Research.

[ref-113] Warwick RM, Platt HM, Somerfield PJ (1998). Free-living marine nematodes. Part III. Monhysterids synopses of the British Fauna, vol. 53.

[ref-114] Wei-Ling N, Cheng-Ann C, Saleem M, Chen-Lin S, Yun-Chih L, Tung-Wei S (2022). Free-living marine nematodes community structure in the conservation area (Chaojing Park) and its adjacent area of Keelung, Taiwan. PLOS ONE.

[ref-115] Wickham H (2016). ggplot2: elegant graphics for data analysis.

[ref-116] Wickham H (2025). https://cran.r-project.org/package=ggplot2.

[ref-117] Wieser W (1953). Die Beziehung zwischen Mundhöhle-Gestalt, Ernährungsweise und Vorkommen bei freilebenden marinen Nematoden. Arkiv för Zoologi.

[ref-118] Wood JR, Wilmshurst JM, Rawlence NJ, Bonner KI, Worthy TH, Kinsella JM, Cooper A (2013). A megafauna’s microfauna: gastrointestinal parasites of New Zealand’s extinct Moa (Aves: Dinornithiformes). PLOS ONE.

[ref-119] WoRMS Editorial Board (2024). World register of marine species. VLIZ. https://www.marinespecies.org.

[ref-120] Yan L (2023). https://cran.r-project.org/package=ggvenn.

[ref-121] Zhao Z, Ye W, Giblin-Davis RM, Li D, Thomas WK, Davies KA, Riley IT (2008). Morphological and molecular analysis of six aphelenchoidoids from Australian conifers and their relationship to *Bursaphelenchus* (Fuchs, 1937). Nematology.

[ref-122] Zhou X, Zeng J, Lizhe K, Fu S, Tan W (2020). Two new species of free-living marine nematodes of the Desmodoridae from mangrove wetlands of Xiamen Bay, China. Journal of Ocean University of China.

