# Supplementary figures and images for: The tip of the iceberg: extraordinarily high diversity while examining two infralittoral nematode communities on Okinawa-jima Island, Japan, using morphology and DNA barcoding
Source: PeerJ. 2025 Jul 30;13:e19757. doi: 10.7717/peerj.19757 (PMC12317692; doi:10.7717/peerj.19757)

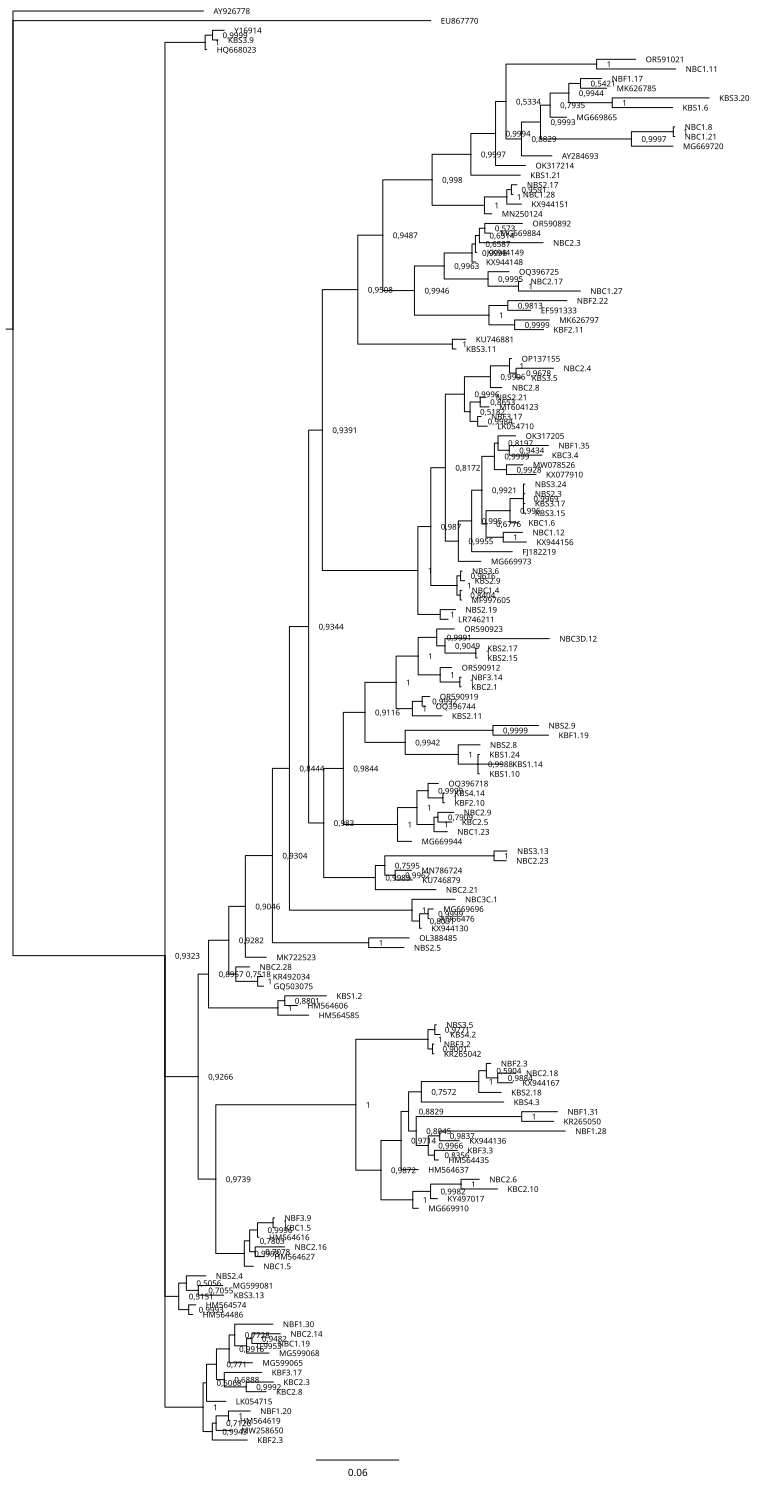

Supplement: Supplemental Information 3 [file peerj-13-19757-s003.png]

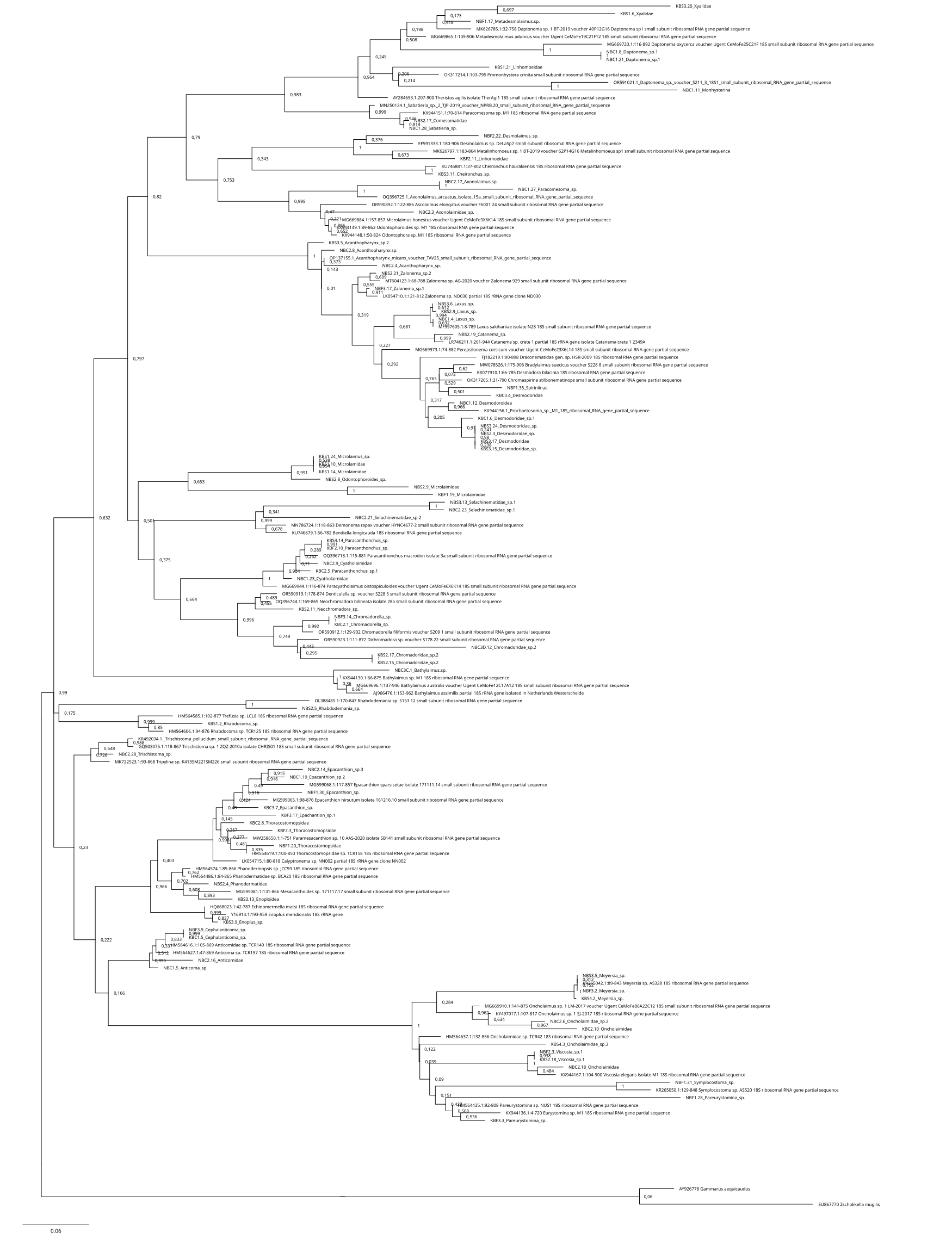

Supplement: Supplemental Information 4 [file peerj-13-19757-s004.png]

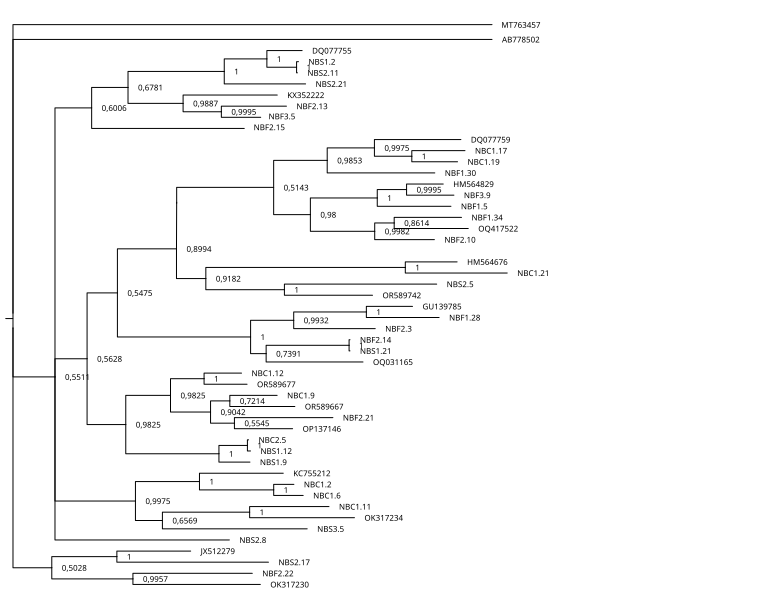

Supplement: Supplemental Information 5 [file peerj-13-19757-s005.png]

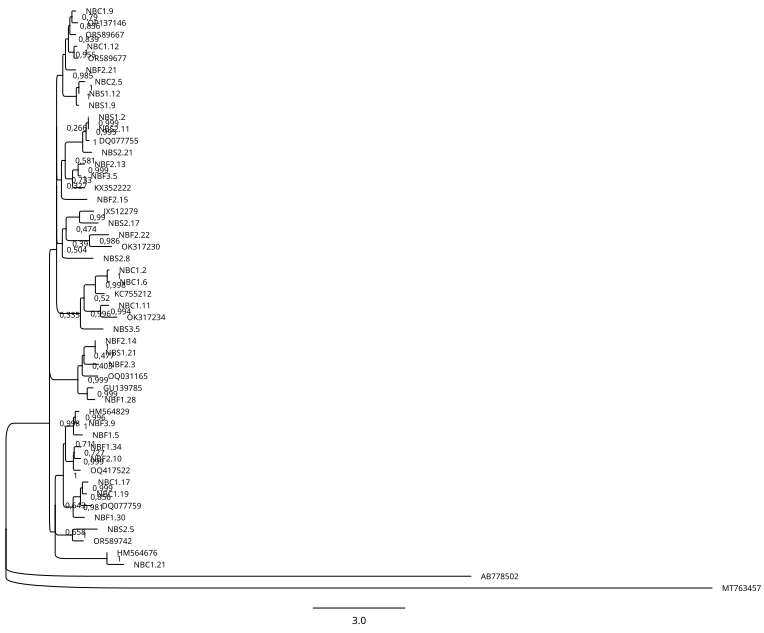

Supplement: Supplemental Information 6 [file peerj-13-19757-s006.png]
