# Supplementary material for: The tip of the iceberg: extraordinarily high diversity while examining two infralittoral nematode communities on Okinawa-jima Island, Japan, using morphology and DNA barcoding
Source: PeerJ. 2025 Jul 30;13:e19757. doi: 10.7717/peerj.19757 (PMC12317692; doi:10.7717/peerj.19757)
Supplement: Supplemental Information 8 [file peerj-13-19757-s008.docx]

| **Paper** | **Authors** | **Year of publication** | **Region** | **Site/s** | **Marker/s** | **Primer pair/s** | **No. of sequences** | **Topology** | **Notes** |
| --- | --- | --- | --- | --- | --- | --- | --- | --- | --- |
| Diversity of free-living marine nematodes (Enoplida) from Baja California assessed by integrative taxonomy | Pereira T. J., et al. | 2010 | Gulf of California and Pacific coast | San Felipe, Santa Clara, Bahía de Los Angeles, La Paz Bay, San Carlos, Faro beach, Cerritos beach | 18S; 28S | MN18F, d22R; D2A and D3B | 139 | 18S gene trees feature 3 clades: Thoracostomopsidae; *Bathylaimus* and other non-identified Enoplida; Oncholaimidae and *Rhabdodemania*. 28S gene trees were less consistent, and in the ML and NJ trees Oncholaimidae and Enchelidiidae were paraphyletic. | Oncholaimidae received the highest bootstrap support in all reconstructions |
| Unraveling free-living marine nematode community structure from a biodiversity-rich tropical coastal setting based on molecular approaches | Kumar A., et al. | 2014 | Central West coast of India | Aagarwadi and Karel | 18S | MN18F/R | 110 | Five orders were found: Enoplida, Desmodorida, Araeolaimida, Chromadorida and Monhysterida. The biggest clade was Tripyloidina (Enoplida), followed by Chromadoridae (Chromadoria). Seven minor clades were found: Xyalidae, Oxystominidae, Cyatholaimidae, Desmodoridae and Leptolaimidae. | This study is the result of the comparison of two libraries |
| DNA barcoding and morphological identification of benthic nematodes assemblages of estuarine intertidal sediments: advances in molecular tools for biodiversity assessment | Avò, A. P., et al. | 2017 | Southwestern coast of Portugal | Mira estuary (4 sites) | 18S rRNA; COI | G18S4-18S_NemR or 18S_NemF-18S_NemR. JB3/JB5 | 114; 60 | From rRNA 18S, sequences clustered into 17 families, of which Xyalidae, Sphaerolaimidae, Comesomatidae, Leptomoeidae, Axonolaimidae, Chromadoridae well supported (ML>75%). Linhomoidae and Oxystominidae were separated into different clades. The tree obtained with COI had lower support values, and only families Axonolaimidae, Anoplostomatidae and Tripyloididae were supported. |  |
| Low endemism, continued deep-shallow interchanges, and evidence for cosmopolitan distributions in free-living marine nematodes (order Enoplida) | Bik H. M., et al. | 2010 | Worldwide | New England (UK), South Africa, California, Bellinghausen Sea, Southern Indian Ocean, and Iberian Margin | 18S; 28S; COI | 18S (SSU_F_04 and SSU_R_26, SSU_F_22 and SSU_R_13, and SSU_F_24_1 and SSU_R_81); 28S (D2A/D3B); COI (JB3/JB5) | 254; 99 for COI | 5 clades were retrieved and named as I, II, III (IIIa,IIIb,IIIc). Phylogenetic relationships amongst Enoplids were congruent in ML and Bayesian trees. Deep-sea species were recovered in most marine families. Deep-sea taxa do not form an independent lineage within Enoplida. | Deep-sea species. phylogenetic structures suggest frequent and recent interchanges between habitats and multiple independent origins for shallow and deep-sea taxa |
| Moving towards a complete molecular framework of the Nematoda: a focus on the Enoplida and early-branching clades | Bik H. M., et al. | 2010 | Worldwide | New England (UK), South Africa, California, Bellinghausen Sea, Southern Indian Ocean, and Iberian Margin | 18S; 28S; COI | 18S (SSU_F_04 and SSU_R_26, SSU_F_22 and SSU_R_13, and SSU_F_24_1 and SSU_R_81); 28S (D2A/D3B); COI (JB3/JB5) | 254; 99 for COI | Enoplia is divided into two orders (Triplonchida and Enoplida) highly supported by ML and Bayesian analyses as monophyletic. Tryplochida contained three sub-clades, and Enoplida had five distinct lineages. In Enoplida, Clade I contained the Rhabdolaimidae, the genera *Syringolaimus* and *Campydora*, Clade II Alaimina and Ironidae (excluding *Syringolaimus*), Clade III Tripyloididae and Trefusiidae, Clade IV Oxystominidae and the superfamily Oncholaimoidea, Clade V some specimens of the superfamily Enoploidea and Leptosomatidae. | Small and large 18S datasets of Enoplid taxa and all nematode taxa found congruent topologies. Certain higher clade relationships within Enoplida remained unresolved. 28S and COI were too variable for inferring deep phylogeny. Many 18S and 28S tree topologies were corresponding at a genus level, fewer possible from COI datasets. |
| Systematics and DNA barcoding of free-living marine nematodes with emphasis on tropical desmodorids using nuclear SSU rDNA and mitochondrial COI sequences | Armenteros M., et al. | 2014 | Cuban Archipelago | Punta Francés Reef | 18S; COI | G18S4/4R; JB3/JB5 | 51; 61 | 18S: 6 clades (Enoplida; Monhysterida; Comesomatidae and Xyalidae; Desmodoridae; Oncholaimidae; *Longicyatholaimus* and *Cheironchus*. Desmodoridae formed a well-supported clade, but subfamilies and genus level taxa were not supported as monophyletic.  COI: 4 clades Xyalidae; Chromadoridae; Cyatholaimidae; Oncholaimidae. Within Desmodoridae the three subfamilies appeared non monophyletic. The ML tree did not provide support for deep relationships in the phylum |  |
| Exploring the use of Cytochrome Oxidase c Subunit 1 (COI) for DNA barcoding of free-Living marine nematodes | Derycke S. et al. | 2010 | Europe | Westerschelde estuary (The Netherlands); Nieuwpoort (Belgium) | COI | JB2s3/JB7GED | 102 | 41 species belonging to 33 genera. No support at a family level was found. | No clade division |
